# Development and Psychometric Properties of a New Questionnaire to Assess Mental Health and Concerning Behaviors in Children and Young People with Autism Spectrum Disorder (ASD): The Assessment of Concerning Behavior (ACB) Scale

**DOI:** 10.1007/s10803-020-04748-1

**Published:** 2020-10-14

**Authors:** Joanne Tarver, Silia Vitoratou, Mathilde Mastroianni, Natalie Heaney, Eleanor Bennett, Felicity Gibbons, Federico Fiori, Michael Absoud, Lakshmi Ramasubramanian, Emily Simonoff, Paramala Santosh

**Affiliations:** 1 Department of Child and Adolescent Psychiatry, Institute of Psychiatry, Psychology & Neuroscience, King’s College London, London, UK; 2 Department of Psychology, School of Life and Health Sciences, Aston University, Birmingham, UK; 3 Department of Biostatistics and Health Informatics, Psychometrics and Measurement Lab, Institute of Psychiatry, Psychology and Neuroscience, King’s College London, De Crespigny Park, London, UK; 4 Centre for Interventional Paediatric Psychopharmacology and Rare Diseases, South London and Maudsley NHS Foundation Trust, London, UK; 5 HealthTracker Ltd, Gillingham, Kent UK; 6 Children’s Neurosciences, Evelina London Children’ Hospital, St Thomas’ Hospital, King’s Health Partners Academic Health Science Centre, London, UK; 7 Department of Women and Children’s Health, Faculty of Life Sciences and Medicine, School of Life Course Sciences, King’s College London, London, UK; 8 Child and Adolescent Mental Health Services, Alder Hey Children’s NHS Foundation Trust, Liverpool, UK

**Keywords:** Autism spectrum disorder (ASD), Emotional and behavioral problems, Risk, Instrument development and validation

## Abstract

Although 70% of autistic children and young people meet criteria for co-occurring psychiatric conditions, there are few screening measures specifically for autistic individuals. We describe the development and validation of the Assessment of Concerning Behavior (ACB), an instrument co-developed with the autistic community to assess mental health and problematic/risky behaviors. Items include descriptions to facilitate symptom recognition by autistic people, and carers/professionals. The ACB was completed by 255 parents, 149 autistic children and young people and 30 teachers. Internal consistency, stability and validity was assessed. The ACB parent-version fit a two-factor model (internalizing and externalizing problems) and showed adequate test–retest reliability, internal consistency and construct validity. The ACB is a promising new measure for research and clinical use in autism.

**Electronic supplementary material:**

The online version of this article (10.1007/s10803-020-04748-1) contains supplementary material, which is available to authorized users.

## Introduction

Autism Spectrum Disorder (ASD) is a neurodevelopmental condition characterized by differences in social communication skills and the presence of restrictive and repetitive behaviors and sensory processing difficulties (APA 2013). Global prevalence rates suggest that around one in 160 children have autism^1^ (Elsabbagh et al. 2012). Emotional and behavioral problems, and co-occurring mental health conditions, are also prevalent in children and young people with autism. Rates of psychiatric conditions generally exceed rates observed in the general population (Joshi et al. 2013; Lai et al. 2019; Salazar et al. 2015); as many as 70% of autistic children and young people will meet criteria for at least one psychiatric condition (Simonoff et al. 2008). Anxiety, depression, oppositional defiant disorder (ODD) and attention deficit hyperactivity disorder (ADHD) are among the most common co-occurring diagnoses (Lai et al. 2019; Russell et al. 2016; Simonoff et al. 2008). Co-occurring symptoms can impact quality of life and lead to poorer long-term outcomes for autistic individuals (Howlin and Magiati 2017; Kuhlthau et al. 2018). Further, they are associated with elevated levels of parent and carer stress (Cadman et al. 2012; Yorke et al. 2018).

In addition to co-occurring psychiatric symptoms, other behaviors and risk factors are common in autistic individuals that are important for consideration during clinical assessment. Examples of such behaviors and risk factors could include: suicidality risks (Hirvikoski et al. 2016); physical health conditions associated with pain, behavior and well-being such as gastrointestinal problems (Mazurek et al. 2013); and concerning social behavior including inappropriate sexual behaviors or vulnerability to exploitation by others (Fisher et al. 2013; Turner et al. 2017). Such behaviors could form important treatment targets or might be indicative of co-occurring conditions. Despite the prevalence of co-occurring conditions and risk behaviors, there are currently few assessment measures available that are targeted specifically for the needs of autistic individuals, or that are valid for use with individuals with intellectual disability (ID) (Flynn et al. 2017).

There are a number of challenges associated with identifying and evaluating co-occurring symptoms in autism. First, multi-informant assessment, including self-report, is the gold standard for diagnosis of mental health conditions; the presence of ID (prevalence approximately 50%; (Charman et al. 2011)) and communication difficulties in autism can complicate the assessment. In individuals with severe to profound ID, obtaining subjective reports of internal states can be near impossible (Adams and Oliver 2011). It can also be difficult for those with IQ in the normal range to self-report. For example, the presence of alexithymia in autism may also impact an individual’s ability to accurately identify and describe their internal state (Shah et al. 2016). Indeed, in a study exploring the parent-reported behavioral manifestations of anxiety in autistic children, items related to a child’s verbal expression of their internal state were endorsed less frequently than items related to observable behavioral manifestations of anxiety (Hallett et al. 2013). Second, mental health conditions can also show an atypical profile in individuals with autism and ID (Ozsivadjian et al. 2012; Reardon et al. 2015); existing mental health measures based on standard diagnostic criteria may not include items suited to the atypical profiles (e.g. atypical phobias or anxiety related to routines and special interests; (Kerns et al. 2014)). Third, mental health symptomology and autism characteristics can overlap. For example, clinicians may find it difficult to delineate restrictive and repetitive interests from compulsions and obsessions more akin to obsessive compulsive disorder (OCD), or social disinterest from social anxiety (Kreiser and White 2014; Zandt et al. 2007). Finally, diagnostic overshadowing means some clinicians may misattribute emotional and behavioral problems to a diagnosis of autism, rather than a co-occurring condition (Rosen et al. 2018).

Failure to identify and assess co-occurring conditions presents a significant health inequality issue for autistic children and young adults, with evidence that a large proportion will meet criteria for a lifetime psychiatric diagnosis in research studies, despite never receiving a clinical diagnosis (Buck et al. 2014). This inequality is especially the case given the growing evidence base that co-occurring conditions in autism (e.g. hyperactivity and anxiety) are amenable to intervention (Arnold et al. 2012; Storch et al. 2013; Wood et al. 2015). In order to ensure the best outcomes for autistic young people and their families, there is a need to develop measures able to accurately detect and assess co-occurring conditions. Further, they should be able to assess change over time and following intervention. In order to meet the needs of the autistic population, measures should also assess potential atypical indicators of co-occurring conditions (Kerns et al. 2014), in addition to other markers of risk that negatively impact autistic individuals, such as social exploitation (Fisher et al. 2013). Self-report versions should also be available for autistic young people who are able to self-report.

Within the autism literature, the Aberrant Behavior Checklist (ABC; (Aman et al. 1985)) and the Developmental Behavior Checklist (DBC; (Einfeld and Tonge, 1995)) are among the most commonly used measures to assess emotional and behavioral problems. The ABC in particular is widely used in autism treatment studies, and shows sensitivity to change as a primary outcome measure in both pharmacological and non-pharmacological treatment studies for emotional and behavioral problems in autistic children (Arnold et al. 2012; Bearss et al. 2015). Further, the factor structure of the ABC has generally been shown to be robust in a sample of autistic children, with 90% of items loading onto originally assigned factors (Kaat et al. 2014). Although overlap between items on the irritability and hyperactivity subscales and a separate self-injury subscale has been reported in another investigation of the ABC with autistic children (Brinkley et al. 2007). Despite the strengths of the ABC and DBC, their intended development and use for those with developmental disabilities means they may be less appropriate for 50% of the autistic population (Charman et al. 2011). For example, assessments of worries and concerns, which are commonly reported in autistic children and young people (Simonoff et al. 2008), are not included in the ABC.

Differing presentations may also be missed by numerous screening and assessment tools originally developed for typically developing (TD) groups. The Child Behavior Checklist (CBCL) (Achenbach and Rescorla 2001b) and the Strengths and Difficulties Questionnaire (SDQ) (Goodman 2001) have extensive evidence of their psychometric properties in TD groups and there is emerging evidence of the psychometric properties of the CBCL (Pandolfi et al. 2009) and SDQ (Findon et al. 2016) in autistic individuals. The Child and Adolescent Symptom Inventory (Gadow and Sprafkin, 1997, 1998) is a DSM-IV referenced scale assessing mental health symptomology also widely used in autism research and treatment studies (Sukhodolsky et al. 2008; White et al. 2009). Whilst evidencing the validity of measures developed for TD groups in autistic populations is important to allow comparison between autistic and other clinical groups, it cannot be certain that these tools measure items of importance to the autistic community. Further, given the limited number of response options on screening tools such as the CBCL and the SDQ (e.g. three response options covering ‘not true’, ‘somewhat true’, ‘certainly true’), and recall period of 6 months, their ability to detect severity and change over short periods of time may be more limited.

The development of measures specifically for autistic individuals is now receiving more attention. For example, The Anxiety Scale for Autism Spectrum Disorder (ASC-ASD; (Rodgers et al. 2016)), and the Parent Rated Anxiety Scale for Youth with Autism Spectrum Disorder (PRAS-ASD; (Scahill et al. 2019)) are two measures for anxiety in autistic children, developed following recognition of the lack of acceptable outcome measures. Kalb et al. (2018) have also reported the development and validation of a parent-reported measure to assess mental health at times of crisis in autistic children and young adults. Despite the clinical importance of measures with a specific focus on areas of mental health, they may be less appropriate to screen for a range of psychiatric comorbidity and symptomology. The Autism Behavior Inventory (ABI) is another recently developed measure designed to assess autism characteristics and includes some items assessing associated behavior including mood and anxiety, self-regulation and challenging behavior (Bangerter et al. 2017, 2019). Although extensive user input was obtained during the development of the ABI, the dual focus on autism characteristics and associated behavior makes it unlikely that the measure will capture the breadth of co-occurring behaviors that can occur in autism. To the best of our knowledge, there are no assessment tools specifically for autistic children and young people, that include self-report measures, and are able to screen for a range of coexisting symptoms and risk behaviors and display sensitivity to change.

This paper describes the development of the ‘Assessment of Concerning Behavior Scale’ (ACB), a new screening measure for concerning behaviors in individuals with autism. The ACB was co-developed with input from the autistic community in order to ensure the inclusion of items considered most important to the autistic population. For the development of this measure and following feedback from our autistic adult and parent advisory panels, the term ‘concerning behavior’ was chosen to describe the behaviors associated with co-occurring conditions, or other markers of risk, important for clinical evaluation and assessment. The objective of this paper is to describe the development of the parent, teacher and self-report scales. In this initial study, we also report the psychometric validation of the parent/carer report version of the ACB in a sample of autistic children and young people, including a confirmatory factor analysis of the proposed factor structure in an independently recruited sample.

## Method

The protocol of the development and validation of the ACB scale has been described previously (Santosh et al. 2016). The study was part of the wider National Institute for Health Research (NIHR) programme grant entitled ‘Improving Autism Mental Health’ (IAMHealth; reference number RP-PG-1211–20,016; https://iamhealthkcl.net). The study received ethical approval from the NHS Research Ethics Committee: London-Camden and King's Cross (ref: 15/LO/0085).

### Development of the ACB Scale

#### Literature Review

Members of the IAMHealth consortium, and clinicians working within specialist mental health services with extensive clinical knowledge in the assessment of autistic individuals (see acknowledgements section), reviewed the literature to identify factors and themes considered to be pertinent for the assessment of concerning behaviors. To ensure that only clinically relevant themes were extracted, papers included in the systematic review were studies in which autistic individuals met diagnostic criteria as outlined by DSM-IV, DSM-V, ICD-10 or a validated measure of autism characteristics, that explored the prevalence of comorbid diagnoses, and studies that assessed this prevalence using DSM-IV, ICD-10 or another validated measure. Following the review of the literature and feedback from the clinical panel, the review led to a list of 220 potential questionnaire items that covered 53 domains of behavior and functioning (Supplementary Table S1).

#### Focus Groups

A total of five focus groups were conducted with autistic children and young people (n = 4), autistic adults (n = 4), parents/carers of individuals with autism (n = 5), clinicians from mental health settings with experience of working with individuals with autism (n = 5), and teachers with experience of working within special educational settings (n = 5). Focus groups started with an open-ended discussion of domains of mental health and concerning behavior participants felt should be included in the questionnaire. Following this, participants were also presented with the 220 draft questionnaire items developed following the literature review and were asked for their feedback on the relevance and importance of the domains and the wording of items. This included seeking participant feedback on how to differentiate concerning behaviors associated with potential psychopathology from behaviors associated with autism characteristics. Domains considered to be less important by focus group participants were dropped from the questionnaire. Feedback was also sought on a range of response scales to be incorporated into the measure. To aid rater understanding of ACB items, focus groups suggested and provided feedback on behavioral examples for each item. For example, an item related to hyperactivity includes examples of excessive running around or often leaving seat when should be sitting down. Behavioral examples were also approved by a panel of experts and particular consideration was given to the wording of items more likely to be affected by symptom overlap. For example, an item designed to assess obsessions and compulsions (item 34) was altered to place emphasis on the removal of distress associated with the behaviors as opposed to behaviors being conducted for pleasure.

Focus group data were transcribed and analyzed using NVivo (QSR International 2008). The aim of the analysis was to identify additional items that had not been captured by the literature review to be added to the item pool, but also to identify other themes evident in participant narratives important for consideration during instrument development. Themes identified were: the need for clear concrete language; the ability to tease apart autism characteristics from co-occurring conditions; need to assess change over time; and the need for a brief tool. Descriptions and example quotes for each theme are provided in Supplementary Table S2.

Once all participant and expert feedback had been incorporated, the beta version of the ACB scale was uploaded onto HealthTracker™, an online health monitoring platform (https://www.healthtracker.co.uk).

#### Beta Version of the ACB

Four versions of the questionnaire were developed for completion by parents and teachers, and self-report (SR) versions for children (aged 7–11; child-SR) and adolescents and young adults (aged 12 and above; YP-SR) were created. Versions of the questionnaire contained the same items, differing only in the wording at the start of the question according to who is responding (e.g. Does your child have nightmares vs. do you have nightmares). However, the child-SR questionnaire did not include items that the expert review panel considered inappropriate to ask young people aged under 12 years (item numbers 6 ‘movements speeded up or slowed down; 17 'trouble with the police’; 19 ‘hard to be happy with self or other people’; 28 'sexual behaviors bother others’; 35 'drugs or alcohol’). The parent-report version included all items, regardless of age of child.

The beta version of the questionnaire included 46 items in the parent, teacher, and YP-SR versions, and 41 items in the child-SR version. Items were rated on a 5-point scale from 0 (not at all) to 4 (very much) according to how much of a problem each behavior had been for the person during the last 1 month. The child-SR and YP-SR questionnaires had Flesch-Kincaid reading grade levels (Flesch 1948) of 3.7 and 4.5, respectively, meaning the questionnaire could be read and easily understood by those with reading ability of U.S. grades 3–4 (ages 8–10).

### Validation of the ACB Scale

#### Initial Sample

For the purposes of this study, parents and carers of children, adolescents and young people with autism were recruited from clinical services and special needs schools (schools providing specialist educational support to children with neurodevelopmental conditions, specific learning needs and/or ID) in the London area. Recruitment was also supplemented with recruitment from a local support group and an existing participant database for parents/carers of autistic children (ASD-UK). Participating clinics were those that provided diagnostic and intervention services (largely around mental health symptomology and behavior) for autistic children and young people. Participants meeting eligibility criteria were invited to take part in the study by clinicians working within clinical services, or from researcher invite following caseload screening. Nine special needs schools in the London area also supported recruitment by sending study invite letters home to parents of children meeting eligibility criteria. In order to ensure a representative sample, very few exclusion criteria were applied to study recruitment. Study invitations were sent to those with a confirmed clinical diagnosis of an ASD (according to school/clinical records) and aged 7 years or older.

**Fig. 1:**
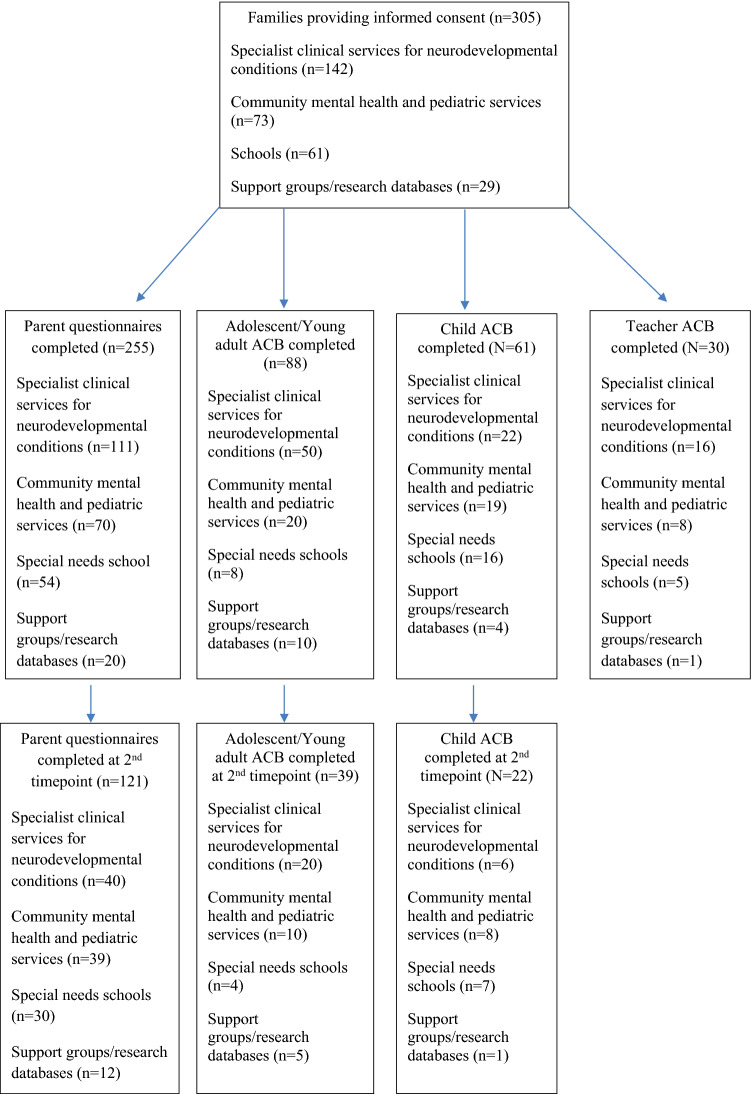
Participant recruitment and questionnaire completion

A total of 305 families were recruited and gave written consent of which 255 parents of autistic children and young adults (*m*_*age*=_12.7 years, *sd* = 3.3, range 7–29) completed the ACB (see Table 1). 149 autistic children and young people also completed the SR version of the questionnaire (Table 1). See Fig. 1 for a flow diagram of participant recruitment.

**Table 1:** Demographics characteristics of initial sample

	Parent report (N = 255)	Teacher report (N = 30)	Young person SR (N = 88)	Child SR (N = 61)
Age (years)				
Mean (SD)	12.8 (3.3)	11.5 (2.7)	15.1 (2.5)	9.9 (1.5)
Min–max	12.8 (7–29)	11.3 (5–16)	14.6 (12–29)	9.9 (8–14)
Sex				
Male	193 (75.7%)	24 (80%)	66 (75%)	51(83.6%)
Female	58 (23.1%)	6 (20%)	21 (23.9%)	9 (14.8%)
Not selected	4 (1.6%)	–	1 (1.1%)	1 (1.6%)
Ethnicity				
Asian	7 (2.7%)	1 (3.3.%)	1 (1.1%)	-
Black	14 (5.5%)	1 (3.3%)	4 (4.5%)	5 (8.2%)
White	216 (84.7%)	26 (86.7%)	76 (86.4%)	53 (86.9%)
Mixed or other	18 (7.1%)	2 (6.7%)	7 (8%)	3 (4.9%)
SCQ score				
Mean (SD)	23.1 (7.5)	19.5 (6.3)	21.1 (7.2)	23.4 (7.7)
Median (min–max)	23 (3–38)	21 (7–32)	22 (3–36)	25 (7–36)
Developmental quotient				
Mean (SD)	67.3 (27.2)	74.8 (31.2)	77.8 (23.9)	73.1 (27)
Median (min–max)	68.11 (12.5–182.61)	73.58 (36–183)	80.27 (13.0–131.5)	73.58 (13–183)

### Procedure

The research team made contact with eligible participants via telephone or email to describe the study in detail and invite participants to take part. Consent forms were posted to eligible participants who provided initial verbal consent during the initial contact. Upon receipt of completed participant consent forms, questionnaires were sent to participants to complete online or paper copies to be returned in prepaid envelopes.

Separate log-in details were provided for parents/carers and autistic individuals completing SR versions of the questionnaire. Children and young people who had a documented ID, or recruited from learning disability services, were not invited to complete SR questionnaires. Other than the presence of ID, the main reasons (according to parent-report) for not completing the SR questionnaires included non-compliance, poor attention span or performance-related anxiety or distress related to questionnaire completion. Given that SR questionnaires were also completed at home, parents were asked to support the autistic individual if necessary, but not to influence the child/young person’s responses to the questionnaire. With parental consent, teachers were also invited to complete the ACB. Information sheets and consent forms were posted or emailed to teachers to invite them to take part in the study.

122 parents, 39 young people and 22 children completed the ACB at two timepoints (Fig. 1); the mean durations between completions were 3.05 months (*sd* = 2.23), 2.97 months (*sd* = 1.67) and 2.92 months (*sd* = 2.60) respectively.

### Quest Sample

ACB data collected in a separate research project conducted as part of the IAMHealth programme grant were also used. These data were provided by participants taking part in the Quest follow-up study, a longitudinal cohort of autistic children born between 1^st^ September 2000 and 31st August 2004 and living in two London boroughs. Autism characteristics in the Quest sample were first assessed using the Social Communication Questionnaire Lifetime version (SCQ; (Rutter 2003)). Following this, a multidisciplinary clinical diagnosis for ASD was established, led by a single community pediatrician for each borough. Diagnoses were established using information from multiple sources including parents, teachers, social workers, observation of the child (at clinic or at home and/ or school) and structured assessments such as the Autism Diagnostic Interview-Revised (Lord et al. 1994). For further information on original sampling, see Salazar et al. (2015). In this study, ACB data were available from 210 parents of young people with autism (M_*age*_ = 15.4 years, *sd* = 1.11, range = 13.2–17.9) from the Quest sample (Supplementary Table S3).

### Measures

#### Initial Sample

To characterize the sample, parents/carers of autistic individuals of all ages completed the lifetime version of the Social Communication Questionnaire (SCQ) (Rutter 2003) to measure autism characteristics at the age of 4 to 5 years and currently. The SCQ has shown strong discrimination between autistic and non-autistic cases (sensitivity 0.88, specificity 0.72) (Chandler et al. 2007).

As part of questionnaire batteries, parents/carers were asked to estimate their child’s functioning age (if they felt able) which was used to calculate a developmental quotient (DQ; (estimated functional age/chronological age) × 100). Parent estimated DQ has been shown to correlate highly with scores from formal IQ assessments (Chandler et al. 2016).

#### Measures for Comparison

Parents of autistic children and young people aged 6–18 years completed the Child Behavior Checklist (Achenbach and Rescorla 2001b). The CBCL is a parent-report questionnaire used to detect emotional and behavioral problems in children and adolescents in the past 6 months. Acceptable internal reliability and test re-test has been reported with Cronbach’s alpha ranging from 0.67 to 0.97, and correlations ranging from 0.67 to 0.97 (Achenbach and Rescorla 2001b). The CBCL has been used previously in autism research studies (Mazefsky et al. 2011; Ooi et al. 2011). For autistic individuals aged 18 and above, informants completed the Adult Behavior Checklist (Achenbach and Rescorla 2001a). The ABCL is a 121-item checklist report from a person who knows the adult well. Acceptable test–retest validity has been established (*r* = 0.73–0.94; Achenbach and Rescorla 2001a).

Parents/carers of autistic individuals of all ages completed the Aberrant Behavior Checklist (ABC) (Aman et al. 1985). The ABC contains 58 items that resolve onto five subscales (irritability, lethargy, stereotypy, hyperactivity/noncompliance, and inappropriate speech). Appropriate internal validity (α = 0.86–0.94) and test–retest (*r* = 0.96–0.96) has been reported across the subscales (Aman and Singh 1994). The ABC is widely used in autism treatment studies as an assessment of behavior (Arnold et al. 2012; Bearss et al. 2015).

To assess aggression, parents/carers of autistic individuals of all ages completed the Modified Overt Aggression Scale (Yudofsky et al. 1986). This is a four-part behavior rating scale used to evaluate and document the frequency and severity of aggressive episodes. Inter-rather reliability was found to be acceptable within a sample of adults with ID (ICC = 0.93; (Oliver et al. 2007)).

To assess behavior at home, parents/carers of children (aged below 16) completed the Home Situations Questionnaire (Chowdhury et al. 2016). The HSQ-ASD measures parental reports of behavioral non-compliance in children with autism. HSQ-ASD consists of two 12-item subscales: social inflexible (α = 0.84) and demand specific (α = 0.89). One-week test–retest correlations for social inflexible and demand specific were 0.57 and 0.58 respectively (Chowdhury et al. 2016).

### Statistical Analysis

The first stage of the analysis was to trace potential problematic items using their psychometric properties. The ACB data were reviewed by the panel of experts (senior IAMHealth clinicians with expertise in autism—ES, PS, MA, GB) and VS (statistician) prior to final analyses. Problematic items included items with exceptionally high floor or ceiling effects (items where almost all the responders chose an option at the edge of the rating scale; 0 or 4). These items are less informative as they do not discriminate among participants with different positions on the latent spectrum. Problematic items were also considered items with low stability (test–retest reliability) and items that emerge with very low internal consistency indices. Any problematic items found at this stage were excluded for the analysis. The responses from all four different versions of the questionnaire were studied at this stage.

The panel examined the data and decided to omit certain items based on either low endorsement, low stability, and/or low internal consistency (item numbers: 17 ‘trouble with the police’, 23 ‘stressed or upset about the past’, 30 ‘setting fire to things’, 35 ‘drugs or alcohol’, 37 ‘thinks about killing him/herself’, 38 ‘extremely happy or excited all of the time’ and 44 ‘do things that know shouldn’t to get attention). The final ACB scale consisted of 35 items. The subsequent psychometric analysis was conducted in the parents’ version, where the dataset was large enough to allow for advance statistical models.

### Item Factor Analysis

We conducted a series of Exploratory Factor Analyses (EFA) for categorical items (often referred to as item factor analysis) in the initial sample. Apart from the fit diagnostics and the goodness of fit indices we took under consideration the content validity of the emerging factors. We considered problematic items as items with low loadings (< 0.3) or items with cross-loadings to different factors without clear relation to any of the factors were considered problematic. Next, the EFA suggested model was tested in a second independent sample (Quest Sample) using Confirmatory Factor Analyses (CFA). Exploratory and confirmatory factor analysis were conducted, using the mean and variance adjusted weighted least squares estimator (WLSMVMuthén et al. 1997; Wirth and Edwards 2007)) in the initial and Quest samples respectively. To evaluate the overall model fit in all cases, measures of absolute and relative fit were used, namely: the relative chi-square (χ^2^/df: values close to 2 indicate close fit; (Hoelter 1983)), the Root Mean Square Error of Approximation (RMSEA, values less than 0.8 are required for adequate fit; (Browne and Cudeck 1992)), the Tucker–Lewis Index (TLI, values higher than 0.9 are required for close fit; (Bentler and Bonett 1980)) and the Comparative Fit Index (CFI, values higher than 0.9 are required for close fit; (Bentler 1990)). The Mplus software (Muthén and Muthén 1998) was used in all latent trait analysis.

### Reliability, Validity, and Hypothesis Testing

Reliability and validity were estimated for the final solution (emerged factor structure), for each factor separately. With respect to the reliability of the final scale, its internal consistency was evaluated via Cronbach (1951) alpha coefficient, along with the item-total correlations (ITC) and the computation of the alpha if the item was deleted (AID). Stability was evaluated via Cohen’s weighted Kappa (Cohen 1968) for each ordinal categorical item, following Landis and Koch, (1977) recommendations, and with the percentage of agreement. For the total scores, which unlike the items are continuous variables, the (two-way mixed) intraclass correlation coefficient (ITC; (Shrout and Fleiss 1979)) was calculated. Parametric tests (Pearson correlation coefficients, t-test and one-way ANOVAs) were used in validity and hypothesis testing. Differences in the scores due to age and gender are also presented. Stata software (StataCorp 2017) was used for this part of the analysis. Correlation co-efficients were compared according to Zou (2007) method using the cocor package in R software (Diedenhofen and Musch 2015).

Given the wide range in IQ and functioning level that is seen in autistic groups, descriptive indices, reliability and validity were also explored separately according to DQ level. Participants were grouped according to whether parents reported DQ < 70, DQ ≥ 70 or were unable to estimate DQ for their child. To compare the strength of validity correlations across DQ groups, calculations of correlation difference were also conducted (Eid et al. 2011).

## Results

### Item Evaluation-Frequencies and Percentages

#### Parent Version

The parent version was completed by 255 parents/carers (Table 1). For the majority of items in the parents’ version, most of the responders chose the option ‘not at all’ (see Supplementary Table S4; mode = 0 for most items) and the rest of the responses were almost evenly distributed amongst the response options. For some items, almost all parents selected the first option (0 not at all; 17: Trouble with the police, 28: Sexual behaviors bother others, 30: Setting fire to things, 32: Enjoy hurting people or animals, 35: Drugs or alcohol, 36: See or hear things that others cannot, 40: Control people, 43: Dislike being separated from certain people). On the contrary, most people replied positively (“much” or “very much”) to items 12 (Mood changes very quickly) and 16 (Short attention span). Only 30% of the parents responded to item 37b, which was an item that only appeared if it was reported that the person had suicidal thoughts (item 37a). Supplementary Tables S4 and S5 present the descriptive indices per item for each version.

#### Teacher Version

Thirty teachers completed the ACB (Table 1). In most of the items the teachers selected the option “not at all” (See Supplementary Table S4; mode = 0 for most items). For some items in particular, more than 95% of the responses were “not at all” (for example items 3: Nightmares, 9: Do acceptable things on the internet, 17: Trouble with the police, 28: enjoy hurting people or animals, 30: Setting fire to things, 31: Worry about getting fat, 32: Enjoy hurting people or animals, and 35: do some of your pupil’s senses (hearing, smelling, touching, seeing, tasting) seem to bother him/her). Only 4 teachers responded to item 37a (regarding suicidal thoughts).

#### Young Person SR

Eighty-eight adolescents and young people (aged 12–29; Table 1) completed the YP-SR. Almost identically to the parent version, for most items, most of the participants chose the option “not at all” (1) and the rest of the responses were almost evenly distributed amongst response options (See Supplementary Table S5; mode = 0 for most items). For some items almost all participants selected the first option (“not at all’; 17, 28, 30, 32, 35, 36, 40, and 43) whereas the reverse happened to items 12 and 16. In item 37b only 30% of the participants responded.

#### Child SR

Sixty-one children aged (7–11; Table 1) completed the child-SR. The majority of the children chose the option “not at all” in most items and the rest of the responses were almost evenly distributed to the rest of the response options (See Supplementary Table S5; mode = 0 for most items). Especially for items 9 (Do not acceptable things on the internet) and 30 (Setting fire to things) the vast majority responded, “not at all”. On the contrary, items 16 (Short attention span), 25 (Refuse to follow rules), and 29 (Scared of animals or situations), were frequently endorsed by children with most responses in the “much” or “very much” options.

#### Reliability of the Four Versions

Retest data were available for the parents (*n* = 121), YP-SR (*n* = 39) and child-SR (*n* = 22) versions. The item level stability in time for parents’ and SR versions are presented in Supplementary Table S6.

In terms of test–retest reliability, the results for the parents’ version were satisfactory for all items, with the percentage of agreement exceeding 85% in all cases. During the internal consistency assessment, it became apparent that item 23 (Stressed or upset about past) and item 35 (Drugs or alcohol) were inversely rated by the parents (lower ratings in these items corresponded to higher ratings to the rest of the items).

No data were available for the stability of the teacher version. Regarding the internal consistency, the items 4 (Things that likes to repeat), 10 (Hurt or injure), and 44 (Do things that knows shouldn't, to get attention) were inversely rated by the teachers (lower ratings in these items corresponded to higher ratings to the rest of the items).

The test–retest reliability analyses showed that the results for the YP-SR version were satisfactory for all items, with the percentage of agreement exceeding 85% in all cases. During the internal consistency assessment, it became apparent that the items 17 (Trouble with the police), and 39 (Hard to wake up, sleepy during the day) were inversely rated by the participants (lower ratings in these items corresponded to higher ratings to the rest of the items). The stability of the child-SR version was less satisfactory with 24 of 41 items showing a percentage of agreement of less than 85% (six items had less than 80%; see Supplementary Table S6).

#### Full psychometric assessment of the parent version

Sufficient sample size permitted a full psychometric assessment to be conducted for the parent version of the ACB.

#### Exploratory Factor Analysis

The exploratory factor analysis (EFA) included the refined item list following the expert review. Nine factors met Kaiser’s criterion for eigen value > 1 (11.9, 3.7, 2.7, 1.8, 1.6, 1.5, 1.2, 1.1, and 1.1) and were extracted from the data. All solutions from the univariate to the nine factor solutions were fitted. Satisfactory fit indices (good fit) were first achieved at the two-factor solution. Table 3 presents the goodness of fit indices for the 1, 2 and 3-factor solutions. The one factor solution was not satisfactory. The two-factor solution had close fit yet led to four items (2, 4, 11, and 41) with low loadings at both factors (cross loadings). When the number of factors was increased to three, the loadings of the four items were not improved, and in addition the third factor included only two items. Based on these results the four items were omitted from the current version of the questionnaire as problematic.

EFA was repeated for the remaining 35 items. The two-factor model provided close fit to the data (Table 2) and no problematic loadings emerged (Table 3). The two factors presented a clear distinction between the internalizing and externalizing symptoms. As in the case of the 39 items, the 3-factor solution led to low loadings and extensive cross loadings across factors, for a small improvement in the overall fit (Supplementary Table S7). As the characteristics of the two-factor solution were satisfactory across all criteria (content, fit measures, and loadings magnitude) the 35-item 2-factor solution was considered as the final version.

**Table 2:** Goodness of fit indices per model (solution)- EFA (initial) and CFA (Quest) samples (parent version)

Method (sample)	Solution	χ^2^/df	RMSEA (p-close)	TLI	CFI
EFA	1-factor—39 items	2.4	0.074 (< 0.001)	0.80	0.81
EFA	2-factors—39 items	1.7	0.053 (0.158)	0.90	0.91
EFA	3-factors—39 items	1.4	0.040 (0.998)	0.94	0.95
EFA	1-factor—35 items	2.6	0.079 (< 0.001)	0.79	0.80
EFA	2-factors—35 items	1.7	0.053 (0.229)	0.91	0.92
EFA	3-factors—35 items	1.5	0.044 (0.923)	0.95	0.93
CFA	1-factor—35 items	2.0	0.067 (< 0.001)	0.83	0.83
CFA	2-factors—35 items	1.7	0.057 (0.035)	0.87	0.88

**Table 3:** EFA loadings and reliability indices – parents’ version (total: a = 0.90, ICC = 0.67)

Item	EFA (CFA) loadings*		Internal consistency**		Test retest	
	IF	EF	ITC	AID	% agreement	Cohen’s weighted Kappa
8. Spend a lot of the day worried	0.78 (0.7)		0.7	0.9	92.7	0.72
15. Scared when people that don’t know	0.77 (0.63)		0.7	0.9	91.3	0.71
27. Aches, pains and/or lack energy	0.67 (0.58)		0.6	0.9	88.1	0.53
5. Dislike him/herself	0.67 (0.53)		0.6	0.9	91.9	0.66
20. Thoughts and beliefs which are not real	0.65 (0.74)		0.6	0.9	92.7	0.55
19. Hard to be happy with self or other people	0.63 (0.74)		0.7	0.9	88.2	0.57
34. Ritual that must do to stop feeling upset	0.61 (0.45)		0.5	0.9	89.9	0.56
31. Worry about getting fat	0.61 (0.5)		0.5	0.9	93.2	0.51
45. Think and behave in set way	0.56 (0.53)		0.5	0.9	89.8	0.62
43. Dislike being separated from certain people	0.55 (0.48)		0.6	0.9	92.7	0.72
36. See or hear things that others cannot	0.55 (0.66)		0.5	0.9	95.5	0.53
3. Nightmares	0.5 (0.68)		0.5	0.9	94.4	0.66
29. Scared of animals or situations	0.48 (0.55)		0.5	0.9	90.5	0.69
25. Senses seem to bother	0.47 (0.63)		0.5	0.9	89.9	0.62
18. Stopped enjoying things or lost interest	0.45 (0.7)		0.5	0.9	90.5	0.51
39. Hard to wake up sleepy during the day	0.44 (0.44)		0.4	0.9	93	0.69
1. Part of body hurts or itches	0.42 (0.56)		0.5	0.9	87	0.48
7. Very interested and think about a lot of time	0.39 (0.53)		0.5	0.9	83	0.42
42. People force to do things that doesn’t want	0.34 (0.5)		0.3	0.9	93.9	0.36
26. Hit or hurt people		0.89 (0.72)	0.7	0.9	92.8	0.72
13. Damage items		0.82 (0.76)	0.8	0.9	93.3	0.73
24. Refuse to follow rules		0.81 (0.77)	0.8	0.9	91.8	0.72
46. Does things without thinking		0.73 (0.61)	0.7	0.9	88.8	0.64
32. Enjoy hurting people or animals		0.73 (0.74)	0.5	0.9	99.2	0.84
21. Shout at or threaten		0.71 (0.64)	0.7	0.9	91.1	0.69
14. Too much energy		0.62 (0.49)	0.5	0.9	92.7	0.77
12. Mood changes very quickly	0.34	0.62 (0.81)	0.7	0.9	92.5	0.74
40. Control people		0.62 (0.67)	0.6	0.9	88.6	0.58
22. Does not care to upset		0.58 (0.46)	0.6	0.9	88.2	0.57
16. Short attention span		0.51 (0.64)	0.5	0.9	92	0.68
10. Hurt or injure		0.46 (0.56)	0.6	0.9	92.9	0.68
33. Eat too much		0.44 (0.61)	0.5	0.9	94.3	0.8
9. Do not acceptable things on the internet		0.4 (0.49)	0.4	0.9	97	0.49
6. Movements speeded up or slowed down		0.36 (0.59)	0.5	0.9	88.2	0.37
28. Sexual behaviors bother others		0.34 (0.47)	0.3	0.9	96.2	0.53

*IF* internalizing factor: a = 0.86, ICC = 0.80; *EF* externalizing factor: a = 0.88, ICC = 0.83

*Loadings smaller than 0.3 are not presented, **Computed within each factor

### Confirmatory Factor Analysis

The second sample (Quest sample) was used to confirm the factor structure that emerged from the EFA. The one factor solution did not provide adequate fit in the data. The EFA-suggested the two-factor structure had close fit in the Quest data according to the relative chi –square and the RMSEA (Table 3), even though the TLI/CFI values were lower than 0.90. The reason for this inconsistency is the fact that there are items with relative lower loadings in the questionnaire (Table 4 and Supplementary Table S7), which were retained due to their important clinical content.

**Table 4:** Pearson correlation coefficients between the ACB- parent scores and other measures–concurrent, convergent & discriminant validity

	ACB internalizing	ACB externalizing	Total ACB
ACB (N = 255)			
Externalizing	0.50		
Total ACB	0.87ª	0.88ª	
ABC (N = 193)			
Irritability	0.54ª	0.80^b†^	0.76
Lethargy	0.60ª^†^	0.43^b^	0.59
Stereotypy	0.27ª	0.42^b^	0.39
Hyperactivity	0.33ª	0.77^b†^	0.62
Speech	0.25ª	0.28^a^	0.31
Total ABC	0.55ª	0.77^b^	0.75^†^
MOAS (N = 217)			
Verbal aggression	0.26^a^	0.52^b†^	0.44
Aggression against property	0.25ª	0.60^b†^	0.48
Auto aggression	0.28ª	0.46^b†^	0.42
Physical aggression	0.15*,ª	0.56^b†^	0.40
Total MOAS	0.29ª	0.65^b†^	0.53
ASEBA (N = 211)			
Total ASEBA	0.75ª	0.73^a^	0.84^†^
ASEBA internalizing	0.78^a†^	0.38^b^	0.68
ASEBA Externalizing	0.46^a^	0.83^b†^	0.76
SCQ (N = 238)			
Total SCQ	0.25ª^,#^	0.30^a#^	0.32
HSQ (N = 187)			
Social Flexibility	0.44ª	0.64^b†^	0.62
Demand Specific	0.43ª	0.70^b†^	0.65
Total HSQ	0.46ª	0.70^b†^	0.66

*All coefficients were statically significant with p < 0.001 apart from this coefficient where p = 0.024

^†^Moderate to large correlations expected as indicator for convergent validity

^#^Small correlations expected as indicator for discriminant validity

a,b: coefficients that do not share the same subscript within row were significantly different according to Zou (2007) test for overlapping correlation coefficients

### Reliability and Validity

Table 3 also presents the loadings (Geomin rotation) and the within-factors reliability (internal consistency and stability) for the final version. All indices indicated satisfactory reliability, on item and factor level and sufficient internal consistency per factor.

The concurrent, convergent and discriminant validity of the two-factor solution is supported by the fact several measures are highly correlated with one of the factors and low to moderately with the other, in harmony with the conceptual expectations. As expected, the externalizing subscale of the ACB showed large positive correlations with other measures of externalizing behavior including the irritability and hyperactivity subscales of the ABC. The ACB-externalizing also showed moderate to large correlations with all subscales of the MOAS, a large positive correlation with the ASEBA externalizing subscale and moderate to high correlations with subscales of the HSQ. The ACB-internalizing showed smaller correlations with these measures of externalizing behavior (Table 4). The internalizing subscale of the ACB showed moderate to large positive correlations with other measures of internalizing behavior including the ASEBA internalizing subscale and ABC lethargy subscale (Table 4). The ACB-externalizing showed smaller correlations with these measures of internalizing behavior. The total score of the ACB showed large positive correlations with the total scores of the ABC and ASEBA (Table 4). Discriminant validity is also supported by the relatively low correlations of the ACB scores with the SCQ total score (Table 4).

### Descriptive Indices Based on Sex and Age

Supplementary Table S8 presents the descriptive indices per patient, gender and for the total sample. The internalizing factor differed significantly across sexes, with females scoring higher. No differences between sexes were found with respect to the externalizing factor or the total ACB scores. However, age was significantly correlated with the externalized factor only (*r* = -0.26, *p* < 0.01), but not with the internalized factor (*r* = 0.07, *p* = 0.24) or the total score *r* = -0.1, *p* = 0.13).

### Descriptive Indices, Reliability and Validity Based on Developmental Quotient

Supplementary Table S9 presents descriptive indices and alpha values for the ACB for each DQ group. The ACB-externalizing differed between DQ groups. Autistic individuals with DQ < 70 scored higher on this domain than those with DQ ≥ 70 (25.49 vs. 19.27). There were no other differences on ACB scores between DQ groups. Alpha values were greater than 0.8 for ACB factors in all DQ groups, demonstrating the internal reliability of the ACB across functioning levels.

When considering the validity of the ACB, the majority of correlations for key indicators of convergent validity were comparable across DQ groups. There were no significant differences in correlation strength between DQ groups for relationships between the ACB-externalizing and: the hyperactivity subscale of the ABC; the verbal, property, physical and total aggression subscales of the MOAS; the ASEBA-externalizing; and subscales of the HSQ. The correlation between the ACB-externalizing and the irritability subscale of the ABC was smaller in the DQ < 70 group compared to the DQ ≥ 70 group (*r* = 0.74 and *r* = 0.84, respectively), although correlations were large and in the expected direction in all DQ groups. The correlation between the auto-aggression subscale of the MOAS and ACB-externalizing was smaller in the DQ ≥ 70 group compared to the DQ < 70 and no parent estimate of DQ groups (*r* = 0.29 vs. *r* = 0.53 and 0.57 respectively). There was no difference in correlation magnitude between DQ groups for the ACB-internalizing and lethargy subscale of the ABC and internalizing subscale of the ASEBA. Correlations between the ACB-total and total scores of the ABC were also comparable between DQ groups. The correlation between ACB-total and ASEBA total was slightly larger in the DQ ≥ 70 group compared to DQ < 70 and no parent estimate of DQ (*r* = 0.88 vs. *r* = 0.80 and 0.84 respectively; see Supplementary Table S10). Discriminant validity across DQ groups was also demonstrated with comparable small- moderate correlations between ACB subscales and the SCQ in all DQ groups (Supplementary Table S10).

## Discussion

The present study describes the development of the ACB scale, a new measure of mental health symptoms and concerning behaviors in autistic individuals. The ACB was co-developed utilizing feedback and input from autistic individuals, their parents/carers, teachers and clinicians in order to capture items of most importance to the population. A 46-item questionnaire was developed including parent-report, self-report and teacher-report versions. The frequencies and evidence towards reliability for all four forms of the scale (parent, teacher, YP-SR and child-SR) are presented.

A full psychometric analysis was carried out on the parent ACB. A factor analysis resulted in a 35-item measure with a 2-factor model assessing internalizing and externalizing symptoms. This result was consistent in terms of the data driven EFA and the theory (model) driven CFA. The ACB therefore provides scores for subscales comparable to other commonly used screening measures which provide scores of externalizing and internalizing symptomologies (e.g. CBCL). The ACB is a brief, alternative option for clinicians and researchers using items developed specifically for this population. However, the factor analysis will need to be conducted in larger samples for the self-report and teacher versions. Only reliable items, with sensible endorsement, and content validity were retained in the parent-report scale and used in its full psychometric assessment.

In terms of stability, the test retest reliability was satisfactory for the parent-reported version and young person SR measure. However, the child-SR version (children aged 7–11) was less satisfactory; 24 items showed a percentage agreement of less than 85%. This could be because the child version had a low number of test–retest completions; the finding should therefore be replicated in a larger sample of young children. However, the presence of alexithymia, lower reading ability, and higher levels of inattention found in young autistic children (Lyall et al. 2017) could also explain the less satisfactory reliability on the child self-report. Lower levels of reliability on self-report measures have also been reported in younger children in TD groups (Riley et al. 2004). Other researchers have stated the need to exercise caution in the interpretation of psychiatric self-report measures in children and young people with autism. For example, Mazefsky et al. (2011) found child self-report measures can show high rates of false negatives when predicting diagnoses based on full psychiatric interviews with parents. Despite this, the higher rates of stability found on the young person self-report version of the ACB is a promising finding.

The concurrent validity of the parent-report version of the ACB was also evidenced in this study. The internalizing and externalizing subscales correlated with other measures of concerning behaviors and emotions in the expected direction. The externalizing subscale of the ACB showed large positive correlations with the irritability and hyperactivity subscales of the ABC (*r* = 0.80 and 0.77, respectively) and the externalizing subscale of the ASEBA. The internalizing subscale of the ACB displayed moderate to large positive correlations with the lethargy subscale of the ABC (*r* = 0.60) and the internalizing subscale of the ASEBA (*r* = 0.78). Further, where expected large correlations have been observed (e.g. ACB externalizing and ABC irritability), moderate to low correlations are observed with the alternate ACB subscale. This supports the concurrent, convergent and discriminant validity of the two-factor model of the ACB.

Whilst the ACB shows high correlations with other measures such as the ASEBA and the ABC, the ACB remains a significant contribution to the field. Through its development with the autistic community and validation in this population, the ACB contains items of relevance to the autistic population. For example, items with low endorsement related to drug and alcohol use, involvement with the police and fire setting were removed from this measure. As a result, the ACB is a briefer, 35-item measure assessing both internalizing and externalizing symptomology. Given the high prevalence of both emotional and behavioral conditions in autistic children and young people (Simonoff et al. 2008), there is a need for brief measures of broad constructs of mental health that can be used regularly in research and clinical assessments. Further, validity indicators for the ACB were comparable across the spectrum of functioning in this study; the ACB therefore represents a tool valid for use across the spectrum of autistic children and young people.

Moderate correlations between the internalizing subscale of the ACB and subscales of other measures of externalizing or challenging behavior (e.g. correlation between ACB-internalizing and total score HSQ *r* = 0.46) are supportive of a relationship between emotional problems and challenging behaviors in autism that has been previously reported (Turygin et al. 2013). One possibility is that some autistic children engage in challenging behavior to escape anxiety provoking situations (Egger et al. 2003).

Finally, we conducted analyses to explore relationships between scores on the ACB scales and the sex and age of autistic individuals. We found no differences in parent-reported externalizing behavior on the ACB according to sex, contrary to other studies (Salazar et al. 2015). However, females scored significantly higher on the internalizing subscale compared to males. Again, this is contrary to other studies of emotional and behavioral problems in autistic children where no sex differences were observed (Brereton et al. 2006; Chandler et al. 2016) but in keeping with general population samples where females generally score higher on measures of internalizing behavior (Rutter et al. 2003). However, we were unable to extrapolate the lack of sex differences in ACB-measured externalizing behavior in the recruited sample, this provides an important area for future research.

There are a number of strengths and limitations to this research. The inclusion of autistic individuals, parents, teachers and clinicians in the development of the measure is in-keeping with guidelines from the US Food and Drug Administration for the development of patient reported outcome measures (PROMS; (US Department of Health and Human Services Food and Drug Administration 2006). This inclusion of the autistic community has led to the development of a measure specifically for autistic populations. The main limitation is the low completion rates on the child, young person and teacher versions of the scales. However, a full psychometric analysis was completed for the parent version of the scale during this initial study; parent-report measures are commonly used in both research and clinical services. Future research will need to establish norms and cut-offs for the ACB. Low completion rates amongst children and young people were primarily due to comorbid intellectual disability, non-compliance and living in a residential placement or hospital also contributed. Despite this, the young person self-report version of the ACB still achieved satisfactory test–retest reliability and internal consistency. Another limitation is that participants completed the ACB across a variety of contexts; these include clinical settings, during home visits, on paper versions and online. This could affect the reliability and validity of the results, as perhaps these differing conditions influenced the participants responses. For example, the presence of the researcher during home visit completion may have caused the individual to under- or over-report symptoms. However, statistical analysis showed good reliability and validity across all scales, suggesting that any environmental effect was minimal.

### Future Research

Future research is needed to conduct a full psychometric validation of all versions of the questionnaire. This is particularly important for the self-report measures which are vital for multi-informant assessments, considered the gold-standard for the assessment of mental health. Whilst proxy measures of mental health may be adequate when assessing observable behaviors commonly associated with psychopathology, they are likely to be less reliable for rarer symptoms (e.g. those associated with psychosis; (Adams and Oliver 2011)). Although originally developed for use across the age-span, this initial psychometric validation study focused on autistic children and young adults (aged 7–29); our future objective is to explore the relevance and validity of the ACB with older autistic adults. Further, whilst the ACB was developed for use within autistic populations, the exploration of its use and validity in other populations or clinical groups is also an important area for future research.

## Conclusion

This study describes the development and validation of a new, brief, screening tool to measure mental health and concerning behavior in autistic populations. The ACB is a new measure to provide a quantitative assessment of behavior that could be important treatment targets or indicative of co-occurring conditions in autism warranting further assessment. The involvement of autistic individuals, parents, teachers and clinicians in its development means the ACB includes items of relevance and importance to key stakeholders. All versions of the ACB have been found to be valid and reliable, with satisfactory test–retest reliability and internal consistency. The parent version of the scale was shown to be psychometrically sound, fitting a two-factor model. This indicates that all versions of the ACB are suitable to assess the occurrence of concerning behaviors in autistic children and young people.

## Electronic supplementary material

Below is the link to the electronic supplementary material.

Supplementary file1 (DOCX 81 kb)

## Footnotes

1 We use ‘autism’ to refer to Autism Spectrum Disorder (ASD; DSM-5) and ‘autistic individuals’ as this is the preferred term of some of the autism community (Kenny et al. 2016).
