# Supplementary material for: Development and Psychometric Properties of a New Questionnaire to Assess Mental Health and Concerning Behaviors in Children and Young People with Autism Spectrum Disorder (ASD): The Assessment of Concerning Behavior (ACB) Scale
Source: J Autism Dev Disord. 2020 Oct 14;51(8):2812–28. doi: 10.1007/s10803-020-04748-1 (PMC8254716; doi:10.1007/s10803-020-04748-1)
Supplement: Supplementary file 1 — Supplementary file1 (DOCX 81 kb) [file 10803_2020_4748_MOESM1_ESM.docx]

| **Supplementary Table 1: Proposed domains and items from literature review** |
| --- |
| **Domain 1: Hyperactivity and Impulsivity** |
| Is your child restless during every day routine activities? |
| Is your child unable to remain seated when they should be sitting down? (e.g. in during mealtimes or when completing homework? |
| Does your child constantly fidget with their hands or feet? |
| Is your child overly active, energetic and always seems as if he/she is ‘on the go’? |
| Does your child appear to act without thinking? (E.g. do they blurt out answers to questions before they’ve been completed, interrupt others, butt into games or not wait their turn?) |
| **Doman 2: Inattention** |
| With the exception of any specific interests your child may have, does your child find it difficult to concentrate on every day? |
| With the exception of things that are very important to them, does your child lose items they need for school or everyday activities? |
| Does your child avoid doing tasks which require concentration and mental effort? (e.g. homework) |
| Does your child have a short attention span? (e.g. does he/she fail to finish tasks or projects? Or do they find it hard to concentrate on one thing) |
| **Domain 3: Depression** |
| Have you noticed a loss of interest in things that your child is normally very interested in, without them developing new interests? (e.g. have you noticed a reduction in any pleasurable repetitive activities your child may usually enjoy?) |
| Have you noticed any decline in your child’s self-care? (e.g. not brushing their hair or their teeth when they normally would, taking less pride in their appearance?) |
| Have you noticed that your child has less energy than normal? |
| Does your child want to sleep more than usual? |
| Does your child appear to be sad, low or crying more than normal? |
| Does your child report feeling guilty often? |
| Does your child ever say that they hate themselves? |
| Does your child often blame themselves when bad things happen? |
| Does your child report feeling ugly or disliking the way that they look? |
| Does your child report that people would be better off without them or if they were not there? |
| Have you noticed that your child is talking a lot less than usual or wants to spend more time on his/her own? |
| Have you noticed a significant change in your child's weight and/or appetite? |
| **Domain 4: Suicide Ideation** |
| Does your child ever talk about other people killing themselves? |
| Does your child ever talk about killing themselves or wanting to die? |
| Does your child ever draw pictures of people killing themselves? |
| **Domain 5: Mania** |
| Does your child appear to be abnormally happy or constantly excited for no obvious reason? (E.g. do they laugh hysterically or inappropriately or are they more talkative than usual) |
| Is your child sleeping less but appearing to have lots of energy? |
| Have you noticed that your child is more interested in enjoyable activities than normal? |
| Have you noticed that your child is showing less fear or taking more risks than usual? (E.g. do they seem much more confident in social situations or appear to be taking part in more dangerous activities than usual? |
| **Domain 6: Bipolar** |
| Does your child go between strong mood changes, lasting at least week, for no reason? (e.g. going from being more energetic, happier and excitable, to periods of less energy and low mood?) |
| **Domain 7: OCD** |
| Does your child feel like they need to repeat certain behaviors to prevent bad things from happening and to help reduce any distress they are experiencing? (E.g. frequently washing hands to avoid germs or repeatedly checking doors are locked?) |
| **Domain 8: Psychosis** |
| Does your child ever have beliefs which seem unusual for their age? (e.g. do they think they can control other people, objects or events with their thoughts?) |
| Does your child ever appear to randomly jump between words with no purpose, meaning, direction or flow to what they are saying? |
| **Domain 9: Hallucinations/Delusions** |
| Does your child ever seem distressed or frightened by people/animals that nobody else can see? |
| Does your child ever seem distressed or frightened by voices that nobody else can hear? |
| Does your child ever seem to react as though, or report that they feel like, they are being touched even when no one else is there? |
| Does your child report to hear voices telling him/her to do things? |
| Does your child report that they feel someone or something is out to harm them? |
| **Domain 10: Anxiety** |
| Does your child seem to spend a lot of the day feeling worried or anxious? (when they are worried they may appear to sweat, tremble, report feeling sick, report a fast heartbeat or have trouble sleeping). |
| Does your child often worry that something bad is going to happen to them? |
| Does your child seem to lose sleep due to worrying? |
| Does your child constantly ask for or seem to require a lot of reassurance? |
| Does your child worry about things that are going to happen in the future? |
| Have you noticed a significant change in your child's weight and/or appetite? |
| **Domain 11: Social Anxiety** |
| Does your child appear to be scared in social situations? (when they are worried they may appear to sweat, tremble, report feeling sick or report a fast heartbeat). |
| Does your child ever resist going out in public? |
| Does your child ever appear to be worried or anxious when they know they will be going out in a social situation? |
| Does your child ever worry about social situations after they have happened? |
| Is your child nervous when talking to or in the presence of adults or children they do not know? |
| **Domain 12: Phobias** |
| Does your child have any intense fears? |
| Does your child appear to dread facing, or avoid, certain objects or situations? |
| Is your child scared of or does he/she run away from certain objects or situations when they come across them? |
| **Domain 13: Separation Anxiety** |
| Does your child cry, shout or become distressed when they are separated from a particular person, even when they are at home? (When they are worried they may appear to sweat, tremble, report feeling sick or report a fast heartbeat). |
| Does your child constantly worry that something bad is going to happen to one of their parents or caregiver? |
| Does your child cling to or follow their parent(s)/caregiver around the house? |
| Does your child have nightmares about being apart from their parents/caregiver? |
| **Domain 14: Attachment** |
| Does your child appear to react badly to affection? |
| Does your child not react to affection at all? |
| Does your child never or rarely show affection? |
| Does your child resist physical contact? (E.g. a kiss or hug from parents)? |
| **Domain 15: Catatonia** |
| Have you noticed any increase or decrease in the speed at which your child performs all of their everyday movements? (They may look like they are moving in slow motion or may make sudden random movements with no purpose). |
| Has your child adopted any new postures or stances that they hold for a prolonged period of time? |
| **Domain 16: Alexythymia** |
| Does your child find it difficult to report what emotion they are feeling? (e.g. whether they are sad, happy or angry?) |
| Does your child struggle to understand why or when someone else is angry, sad or happy? |
| **Domain 17: Anhedonia** |
| Does your child enjoy or look forward to watching his/her favorite TV shows or films? |
| Does your child enjoy or look forward to spending time with their family or closest friends? |
| Does your child seem to enjoy their favorite hobbies or past times? (such as any sports they usually enjoy or reading their favorite book)? |
| Does your child enjoy or look forward to eating their favorite meal? |
| Does receiving compliments or praise from others make your child happy? |
| **Domain 18: Sleep** |
| Does your child struggle with getting to sleep at night? |
| Does your child frequently wake up during the night? |
| Does your child often have nightmares or night terrors? (e.g. do they wake up screaming and sweating? Or do they think people are trying to attack them in their sleep)? |
| Does your child sleepwalk? |
| Does your child wake up too early in the morning? |
| Does your child worry about sleeping alone or refuse to go to sleep unless another person is with them? |
| Does your child struggle to wake in the morning for school? |
| Does your child seem very sleepy in the daytime or often fall asleep? |
| **Domain 19: Motor Coordination** |
| Is your child overly clumsy? (e.g. they drop things, trip over, have little control over their movements) |
| **Domain 20: Repetitive Behavior** |
| Does your child perform any repeated movements that do not seem to have any purpose? (e.g. flap their hands, spin objects, rock themselves) |
| Does your child repeat words or whole sentences over and over? |
| **Domain 21: Routines** |
| Does your child insist on sticking to set routines and become upset if they are changed? (such as insisting on the same driving routes to school or the same family seating arrangements during mealtimes)? |
| **Domain 22: Noise sensitivity** |
| Does your child seem distressed or cover his/her ears when they hear loud sounds or particular sounds? |
| Does your child ignore loud noises? |
| **Domain 23: light sensitivity** |
| Do lights or certain colours distress your child? |
| Does your child stare at bright lights or certain objects because of the way they look or the way that they move? |
| **Domain 24: Texture/food sensitivity** |
| Does your child refuse to eat certain foods because of the texture or the way that they taste? |
| Does your child have to eat in a particular way? (at a certain time, place or in set patterns) |
| Does your child often put toys or objects in their mouth (which are not meant to be eaten)? |
| **Domain 25: Smell Sensitivity** |
| Does your child seem distressed, retch, cover their nose or run away from certain smells which do not bother other people? |
| Does your child seem drawn towards or excited by unusual smells? |
| Is your child particularly sensitive to smells in general? (e.g. do they often notice smells that other people do not?) |
| **Domain 26: Touch Sensitivity** |
| Does your child seem especially sensitive to temperature, pain, touches or materials on their skin? |
| Does your child not seem to feel or respond to pain, hard touches or extreme temperatures? |
| Does your child get excited by, or show a need to touch or rub, textures, surfaces, objects or parts of people’s bodies? |
| **Domain 27: Oppositionality** |
| With the exception of when changes are made to routines or when they are banned from their favorite activities, does your child argue with adults? |
| Does your child refuse to follow reasonable requests/rules? |
| Does your child blame other people for their mistakes or misbehavior? |
| Does your child appear to misbehave for attention or to get a reaction out of others? |
| Does your child get angry if things don’t go their way? |
| Does your child avoid or refuse to do chores? |
| Does your child only follow rules when they are threatened with a punishment? |
| **Domain 28: Conduct** |
| Does your child steal things from other people or from shops, even when they understand that stealing is wrong? |
| Does your child wet or soil themselves on purpose for attention or to upset others? |
| Does your child bully, tease or wind up others? |
| Does your child have poor table manners, even if they are capable of eating properly? (e.g. do they throw food around at meal times?) |
| **Domain 29: Emotional Intensity** |
| Does your child cry, wail or moan easily or over small things? |
| When upset, is it always very difficult to calm your child down? |
| Does your child yell and scream a lot for no reason or over small things? |
| Does your child show extreme and ranging responses to small events? |
| Does your child appear to lack control over their emotional responses to events, conversations, interactions, and situations? |
| Does your child appear to have sudden and dramatic changes in mood? |
| **Domain 30: Temper/Irritability** |
| Does your child often lose their temper? |
| Does your child get annoyed by others, events or objects easily? |
| Does your child have temper tantrums? (e.g. do they scream and shout and attack objects or people if they do not get their own way?) |
| **Domain 31: Agitation** |
| Is your child impatient? |
| Does your child appear to have a low tolerance for pain or frustration? |
| Does your child get very angry, very suddenly for unpredictable reasons? |
| Does your child rock, moan, rub the floor or surfaces, pace or perform other behaviors when they are annoyed or distressed? |
| **Domain 32: Verbal Aggression** |
| Does your child shout at people? |
| Does your child threaten to hurt people? |
| Does your child often get into verbal fights with other children (e.g. such as friends or siblings?) |
| **Domain 33: Property Aggression** |
| Does your child damage or destroy items that belong to them or items that belong to other people? |
| Does your child set fire to objects/property? |
| Does your child destroy, or pretend to ‘kill’ or ‘torture’ toys? |
| **Domain 34: Aggression towards others** |
| Does your child hit or hurt other people or animals on purpose? |
| Does your child often get into physical fights with other children? (e.g. such as friends or siblings)? |
| **Domain 35: Aggression intensity** |
| When angry, does your child stay angry for a long time? |
| Is it difficult to calm your child down if they are angry? |
| **Domain 36: Aggression Intensity** |
| Does your child hurt themselves on purpose?( E.g. bang their head, bite, punch or slap themselves, cut their wrists, ingest toxic substances)? |
| **Domain 37: Sexual Behaviors** |
| Does your child ever touch other children or adults inappropriately? |
| Does your child ever ask strangers for relationships or to engage in sexual acts with them? |
| Does your child ever perform sexual acts on themselves in public? (e.g. masturbate or touch themselves inappropriately?) |
| **Domain 38: Discriminative Behaviors** |
| Does your child ever make inappropriate comments to strangers about the way they look? |
| Does your child ever refuse to play or talk to some people because of the way they look? |
| **Domain 39: Peer Relationships** |
| Does your child struggle to make friends? |
| Does your child appear to be ‘left out’ when they are with other children of their age? |
| Do other children seem scared of your child (e.g. siblings or school friends)? |
| **Domain 40: Suicidal Behavior** |
| Does your child have a history of attempting or actively planning suicide? |
| **Domain 41: Bullying** |
| Has your child been bullied or picked on by other children? |
| **Domain 42: Discriminative views** |
| Is your child confused or distressed by people who don’t look like people they are used to seeing? (e.g. do they believe some people do not fit into their idea of how a person should look because of the color of their skin or facial features)? |
| **Domain 43: Abuse** |
| Has your child ever been physically abused by an adult or another child? |
| Has your child ever suffered from neglect by a caregiver or parent? |
| Has your child ever been sexually abused by an adult or another child? |
| **Domain 44: Communication** |
| Is your child able to communicate as well as you would expect for their age? (e.g. are they able to clearly ask you for things they want - if they are at an age where they should be able to?) |
| **Domain 45: Social Performance** |
| Does your child find it difficult to respond appropriately to social interactions? (e.g. understanding different tones of voice or knowing how to behave in certain social situations?) |
| Does your child interfere with, or disrupt, group activities? |
| Does your child stare at other people inappropriately? |
| **Domain 46: Academic** |
| Does your child perform as well academically as you’d expect them to at their age? |
| **Domain 47: Physical Pain** |
| Have you noticed any changes in your child’s behaviour that may indicate physical discomfort or pain? (e.g. do they clutch an area of their body, rock back and forth more than usual, repeatedly hit a part of their body, not use a part of their body)? |
| **Domain 48: Forensic** |
| Is your child interested in criminal or violent acts? |
| Has your child ever been in trouble with the police? |
| **Domain 49: Drug/Alcohol use** |
| Does your child have a history of taking illegal drugs? |
| Does your child have a history of abusing alcohol? (e.g. regularly drinking to the point that they have memory loss or vomit?) |
| **Domain 50: PDA** |
| Does your child feel bad or guilty after they have done something that they shouldn’t have done? |
| Does your child display concern for other people’s feelings? |
| Does your child often deliberately avoid demands and requests? |
| Does your child often tell lies? |
| Does your child often tell complicated or convincing lies to get what they want or to avoid punishment? |
| Does your child purposefully charm people to get what they want? |
| Does your child seem to get pleasure from hurting animals or younger children? |
| **Domain 51: Obsessions** |
| Does your child have any unhealthy obsessions with objects? |
| Does your child have any unhealthy obsessions with other people? |
| **Domain 52: Life events** |
| Has your child experienced any life events they found very upsetting or distressing? (e.g. the loss of a loved one?) |
| **Domain 53: Eating** |
| Does your child make themselves vomit after they eat (i.e. to try to lose weight or reduce their food intake)? |
| Does your child seem obsessed with food, weight and being extremely thin? |
| Does your child try to keep to a very limited diet, try to hide how little they are eating from others, and/or aim to eat much less than they should be eating every day? |
| Does your child use a lot of diet pills, diuretics (e.g. coffee), or laxatives to lose weight or reduce calories they intake? |
| Does your child exercise too much, to lose a lot of weight quickly? |
| Does your child often binge eat or generally eat far too much? |

| **Supplementary Table 2: Thematic analysis of focus group data** | | |
| --- | --- | --- |
| Theme | Description | Example quote |
| Need for clear concrete language, observable examples, and items appropriate for autistic individuals | The need for the questionnaire to be clear and concrete, especially for the self-report version of the measure. Item wording should consider the possibility of literal comprehension of items. | *The literal way of reading that sentence…because the sentence said that ‘I feel that there’s…’, he said that he had answered no because it’s not that he ‘feels’, he ‘knows’* Clinician  *“If I was to read that at a glance, I would say that isn’t suitable because it can be viewed in two ways”* Autistic individual  *“On some questions you may not understand unless you get an example and I think I found this very useful”* Autistic individual  “*it would be important to be more inclusive for patients who have verbal difficulties by actually adapting the questions to be very simple, straightforward, user friendly.”* Clinician  *“and I think that (sleep change) should be picked up because that could be a sign of depression, but she can’t express that to you.”* Parent |
| Need to tease apart autism characteristics from problematic mental health symptomologies | The measure needs to differentiate between characteristics of autism and co-occurring conditions. The measure should also consider that increases or decreases in autistic characteristics might indicate the presence of a co-occurring condition or psychopathology | *“there are plenty of people with sensory sensitivity for whom it’s not particularly a problem…they just have it. So do they have a sensory, you know, a sensory sensitivity or deficiency which causes problems”* Clinician  *“if they’re obsessive but then it’s turning to the point where they’re becoming more anxious”* Teacher  *“The obsession with the Lego because he’s been very miserable lately since leaving* (name of school) *and he doesn’t want to leave his bedroom and he just constantly wants to play with his lego.”* Parent  *“it might be the only thing that you see that they are starting to stim a lot more.”* Parent |
| Need to assess change over time | Discussion of how observable changes from a person’s baseline is important for consideration for assessment of mental health. | *“change in activity levels, change in fluid intake, change in irritability, you know, speech volume, it would be, I mean these are all kind of observable changes.”* Clinician  *“you know just to make it…physically you can see that they…they’re not right, you know, sometimes they might look differently in themselves even, you know?”* Teacher |
| Need for a brief tool | Discussions for the need for the measure to be brief. The measure should not take lots of time to complete in clinical practice, or be so long it may be difficult for autistic individuals or informants to complete. | *“at the beginning I didn’t have to think so much but at the end I had to think more.”* Autistic individual  *“wanting to make a concise screening tool that takes twenty minutes and then actually hit on all the targets”* Clinician  *“So I think for them a more easy screening questionnaire…at assessment and follow on that they just tick…because you can’t ask tier 3 to do a long questionnaire, they just…frankly they don’t have the time.”* Clinician |
| Additional items | Additional items not identified in the literature search were coded as part of the thematic analysis to be added to the item pool for future consideration | Social vulnerability: *“social vulnerability, financial exploitation, people giving out their credit numbers”* Clinician  Somatic complaints: *“what you see in adults is because of lack of emotional literacy and ability to actually be mindful, how they’re feeling, what’s happening, they internalize and somaticize, so the increase in somatic complaints, they visit GP, all sorts of physical issues.”* Clinician  Problematic behavior on the internet: *“also the whole internet related problems* (should be captured).” Clinician |

| **Supplementary Table 3: Demographic characteristics of CFA (Quest) Sample (N=210)** | | |
| --- | --- | --- |
| **Age (years)** | Mean (*sd*) | 15.4 (1.11) |
|  | Min-max | 13.2-17.9 |
| **Sex** | Male | 174 (82.9%) |
|  | Female | 36 (17.1%) |
| **Ethnicity (n=203)** | Asian | 6 |
|  | Black | 55 |
|  | White | 104 |
|  | Mixed or other | 38 |
| **SCQ score** | Mean (*sd*) | 17.61 (7.06) |
|  | Median (min-max) | 18 (3-35) |
| **IQ at 6 years (n=207)** | Mean (sd) | 73.52 (26.57) |
|  | Median (Min-max) | 79 (19-129) |
| **IQ at 13 years (n=63)** | Mean (sd) | 68.25 (31.60) |
|  | Median (Min-max) | 70 (19-129) |

| **Supplementary Table 4: Descriptive items per item: parent and teacher versions** | | | | | | |  |
| --- | --- | --- | --- | --- | --- | --- | --- |
| **ACB item** | **Parents (N=255)** | | | **Teachers (N=30)** | | |  |
|  | ***Mean (sd)*** | ***Mode***  ***(m-M)*** | ***Median (Q1-Q3)*** | ***Mean (sd)*** | ***Mode***  ***(m-M)*** | ***Median***  ***(Q1-Q3)*** |  |
| 1. Part of body that hurts or itches | 1.4 (1.5) | 0 (0-4) | 1 (0-3) | 0.3 (0.8) | 0 (0-3) | 0 (0-0) |  |
| 2. Say bad things to/refuse to talk because of look | 0.7 (1.2) | 0 (0-4) | 0 (0-1) | 0.5 (1.1) | 0 (0-4) | 0 (0-1) |  |
| 3. Nightmares | 0.9 (1.2) | 0 (0-4) | 0 (0-2) | 0.3 (0.5) | 0 (0-2) | 0 (0-0.25) |  |
| 4. Things that likes to repeat | 2.2 (1.6) | 4 (0-4) | 2 (1-4) | 1.2 (1.4) | 0 (0-4) | 0.5 (0-2) |  |
| 5. Dislike him/herself | 1.4 (1.5) | 0 (0-4) | 1 (0-3) | 1.1 (1.4) | 0 (0-4) | 0 (0-2.25) |  |
| 6. Movements speeded up or slowed down | 1.1 (1.4) | 0 (0-4) | 0 (0-2) | 0.4 (0.9) | 0 (0-3) | 0 (0-0) |  |
| 7. Very interested and think about a lot of time | 2.3 (1.5) | 4 (0-4) | 3 (1-4) | 1.2 (1.4) | 0 (0-4) | 1 (0-2.25) |  |
| 8. Spend a lot of the day worried | 1.9 (1.5) | 0 (0-4) | 2 (0-3) | 1.5 (1.4) | 0 (0-4) | 1 (0-3) |  |
| 9. Do not acceptable things on the internet | 0.4 (0.9) | 0 (0-4) | 0 (0-0) | 0.2 (0.4) | 0 (0-1) | 0 (0-0) |  |
| 10. Hurt or injure | 1.0 (1.4) | 0 (0-4) | 0 (0-2) | 0.3 (0.6) | 0 (0-2) | 0 (0-0.25) |  |
| 11. Changes in mood that last | 1.0 (1.3) | 0 (0-4) | 0 (0-2) | 1.0 (1.4) | 0 (0-4) | 0 (0-2) |  |
| 12. Mood changes very quickly | 2.5 (1.5) | 4 (0-4) | 3 (1-4) | 1.3 (1.5) | 0 (0-4) | 1 (0-2.25) |  |
| 13. Damage items | 1.5 (1.5) | 0 (0-4) | 1 (0-3) | 0.7 (1.0) | 0 (0-3) | 0 (0-1.25) |  |
| 14. Too much energy | 1.7 (1.6) | 0 (0-4) | 1 (0-4) | 1.1 (1.5) | 0 (0-4) | 0 (0-2.25) |  |
| 15. Scared when people that don’t know | 2.2 (1.6) | 4 (0-4) | 2 (1-4) | 1.1 (1.3) | 0 (0-4) | 1 (0-2) |  |
| 16. Short attention span | 2.6 (1.3) | 4 (0-4) | 3 (2-4) | 1.9 (1.4) | 0 (0-4) | 2 (0.75-3) |  |
| 17. Trouble with the police | 0.1 (0.6) | 0 (0-4) | 0 (0-0) | 0.1 (0.4) | 0 (0-2) | 0 (0-0) |  |
| 18. Stopped enjoying things or lost interest | 0.9 (1.3) | 0 (0-4) | 0 (0-2) | 0.6 (1.2) | 0 (0-4) | 0 (0-1) |  |
| 19. Hard to be happy with self or other people | 1.7 (1.5) | 0 (0-4) | 2 (0-3) | 1.2 (1.5) | 0 (0-4) | 0.5 (0-2) |  |
| 20. Thoughts and beliefs which are not real | 0.8 (1.2) | 0 (0-4) | 0 (0-1) | 0.5 (1.1) | 0 (0-4) | 0 (0-0.25) |  |
| 21. Shout at or threaten | 1.3 (1.5) | 0 (0-4) | 1 (0-3) | 0.9 (1.4) | 0 (0-4) | 0 (0-1.25) |  |
| 22. Does not care to upset | 1.7 (1.5) | 0 (0-4) | 2 (0-3) | 1.2 (1.3) | 0 (0-4) | 1 (0-2) |  |
| 23 Stressed or upset about past | 1.6 (1.6) | 0 (0-4) | 1 (0-3) | 1.0 (1.2) | 0 (0-4) | 1 (0-2) |  |
| 24. Refuse to follow rules | 2.2 (1.5) | 4 (0-4) | 2 (1-4) | 1.5 (1.5) | 0 (0-4) | 1 (0-3) |  |
| 25. Senses seem to bother | 2.6 (1.5) | 4 (0-4) | 3 (2-4) | 1.2 (1.6) | 0 (0-4) | 0 (0-2) |  |
| 26. Hit or hurt people | 1.2 (1.4) | 0 (0-4) | 1 (0-2) | 0.7 (1.3) | 0 (0-4) | 0 (0-1) |  |
| 27. Aches, pains and/or lack energy | 1.3 (1.5) | 0 (0-4) | 1 (0-3) | 0.4 (0.9) | 0 (0-3) | 0 (0-0.25) |  |
| 28. Sexual behaviors bother others | 0.3 (0.9) | 0 (0-4) | 0 (0-0) | 0.1 (0.6) | 0 (0-3) | 0 (0-0) |  |
| 29. Scared of animals or situations | 2.1 (1.5) | 4 (0-4) | 2 (1-4) | 1.0 (1.3) | 0 (0-4) | 1 (0-1.25) |  |
| 30. Setting fire to things | 0.3 (0.8) | 0 (0-4) | 0 (0-0) | 0.0 (0.2) | 0 (0-1) | 0 (0-0) |  |
| 31. Worry about getting fat | 0.6 (1.2) | 0 (0-4) | 0 (0-1) | 0.2 (0.8) | 0 (0-4) | 0 (0-0) |  |
| 32. Enjoy hurting people or animals | 0.2 (0.7) | 0 (0-4) | 0 (0-0) | 0 .0(0.2) | 0 (0-1) | 0 (0-0) |  |
| 33. Eat too much | 1.3 (1.5) | 0 (0-4) | 0 (0-2) | 0.5 (1.2) | 0 (0-4) | 0 (0-0) |  |
| 34. Ritual that must do to stop feeling upset | 1.2 (1.4) | 0 (0-4) | 1 (0-2) | 0.3 (0.7) | 0 (0-2) | 0 (0-0.25) |  |
| 35. Drugs or alcohol | 0.1 (0.4) | 0 (0-4) | 0 (0-0) | 0.0 (0.0) | 0 (0-0) | 0 (0-0) |  |
| 36. See or hear things that others cannot | 0.4 (0.9) | 0 (0-4) | 0 (0-0) | 0.3 (0.9) | 0 (0-4) | 0 (0-0) |  |
| 37.Think about killing him/herself | 0.5 (1.0) | 0 (0-4) | 0 (0-0) | 0.2 (0.5) | 0 (0-2) | 0 (0-0) |  |
| 37b.Has tried to kill him/herself | 0.7 (1.2) | 0 (0-4) | 0 (0-1) | 0.5 (0.6) | 1 (0-1) | 0.5 (0-1) |  |
| 38. Extremely happy or excited all the time | 0.9 (1.3) | 0 (0-4) | 0 (0-1) | 0.4 (0.7) | 0 (0-3) | 0 (0-1) |  |
| 39. Hard to wake up sleepy during the day | 1.2 (1.5) | 0 (0-4) | 0 (0-2) | 0.6 (1.2) | 0 (0-4) | 0 (0-1) |  |
| 40. Control people | 1.2 (1.5) | 0 (0-4) | 0 (0-3) | 0.7 (1.3) | 0 (0-4) | 0 (0-1) |  |
| 41. Look after self, less | 0.8 (1.3) | 0 (0-4) | 0 (0-1) | 0.2 (0.8) | 0 (0-4) | 0 (0-0) |  |
| 42. People force to do things that doesn’t want | 0.3 (0.8) | 0 (0-4) | 0 (0-0) | 0.3 (0.6) | 0 (0-2) | 0 (0-0) |  |
| 43. Dislike being separated from certain people | 1.5 (1.5) | 0 (0-4) | 1 (0-3) | 0.6 (0.9) | 0 (0-4) | 0 (0-1) |  |
| 44. Do things that knows shouldn't, to get attention | 1.4 (1.4) | 0 (0-4) | 1 (0-2) | 1.1 (1.2) | 0 (0-4) | 1 (0-2) |  |
| 45. Think and behave in set way | 2.2 (1.4) | 4 (0-4) | 2 (1-4) | 1.1 (1.2) | 0 (0-4) | 1 (0-2) |  |
| 46. Does things without thinking | 2.2 (1.5) | 4 (0-4) | 2 (1-4) | 1.5 (1.5) | 0 (0-4) | 1 (0-3) |  |
| *item not included in child version, m=min, M=Max, Q1=25%, Q3=75% percentiles | | | | | | | |
|  | | | | | | | |

| **Supplementary Table 5: Descriptive items per item: young adult & adolescents’ and children’s versions** | | | | | | |  |
| --- | --- | --- | --- | --- | --- | --- | --- |
| **ACB item** | **Adolescent and young adults (N=88)** | | | **Child (N=61)** | | |  |
|  | ***Mean (sd)*** | ***Mode***  ***(m-M)*** | ***Median (Q1-Q3)*** | ***Mean (sd)*** | ***Mode***  ***(m-M)*** | ***Median***  ***(Q1-Q3)*** |  |
| 1. Part of body that hurts or itches | 1.3 (1.4) | 0 (0-4) | 1 (0-2) | 1.3 (1.4) | 0 (0-4) | 1 (0-2) |  |
| 2. Say bad things to/refuse to talk because of look | 0.4 (1) | 0 (0-4) | 0 (0-0) | 0.6 (1.1) | 0 (0-4) | 0 (0-1) |  |
| 3. Nightmares | 0.8 (1.2) | 0 (0-4) | 0 (0-1) | 1.1 (1.3) | 0 (0-4) | 0 (0-2) |  |
| 4. Things that likes to repeat | 2 (1.4) | 2 (0-4) | 2 (1-3) | 2.2 (1.5) | 4 (0-4) | 2 (1-4) |  |
| 5. Dislike him/herself | 1.2 (1.3) | 0 (0-4) | 1 (0-2) | 0.8 (1.2) | 0 (0-4) | 0 (0-2) |  |
| 6. Movements speeded up or slowed down | 1 (1.3) | 0 (0-4) | 0 (0-2) | 1.8 (1.6) | 0 (0-4) | 2 (0-3.5) |  |
| 7. Very interested and think about a lot of time | 2 (1.5) | 0 (0-4) | 2 (1-3) | 1.3 (1.3) | 0 (0-4) | 1 (0-2) |  |
| 8. Spend a lot of the day worried | 1.6 (1.5) | 0 (0-4) | 1 (0-3) | 0.2 (0.6) | 0 (0-4) | 0 (0-0) |  |
| 9. Do not acceptable things on the internet | 0.3 (0.8) | 0 (0-4) | 0 (0-0) | 0.7 (1.2) | 0 (0-4) | 0 (0-1) |  |
| 10. Hurt or injure | 0.8 (1.1) | 0 (0-4) | 0 (0-1) | 0.7 (1.1) | 0 (0-4) | 0 (0-1) |  |
| 11. Changes in mood that last | 1.1 (1.4) | 0 (0-4) | 0 (0-2) | 2.4 (1.5) | 4 (0-4) | 2 (1-4) |  |
| 12. Mood changes very quickly | 2.1 (1.4) | 2 (0-4) | 2 (1-3) | 0.8 (1.3) | 0 (0-4) | 0 (0-1) |  |
| 13. Damage items | 0.9 (1.3) | 0 (0-4) | 0 (0-2) | 2.2 (1.7) | 4 (0-4) | 2 (0-4) |  |
| 14. Too much energy | 1.7 (1.6) | 0 (0-4) | 1 (0-3) | 2.1 (1.5) | 1 (0-4) | 2 (1-4) |  |
| 15. Scared when people that don’t know | 2.2 (1.5) | 4 (0-4) | 2 (1-4) | 2.4 (1.5) | 4 (0-4) | 2 (1-4) |  |
| 16. Short attention span | 2.3 (1.4) | 3 (0-4) | 3 (1-4) | 0.6 (1) | 0 (0-4) | 0 (0-1) |  |
| 17. Trouble with the police | 0.2 (0.7) | 0 (0-4) | 0 (0-0) | 0.6 (1.2) | 0 (0-4) | 0 (0-1) |  |
| 18. Stopped enjoying things or lost interest | 1 (1.3) | 0 (0-4) | 0 (0-2) | 1.2 (1.4) | 0 (0-4) | 1 (0-2) |  |
| 19. Hard to be happy with self or other people | 1.4 (1.3) | 0 (0-4) | 1 (0-2) | 1.2 (1.4) | 0 (0-4) | 1 (0-2) |  |
| 20. Thoughts and beliefs which are not real | 0.9 (1.4) | 0 (0-4) | 0 (0-2) | 1.4 (1.5) | 0 (0-4) | 1 (0-3) |  |
| 21. Shout at or threaten | 1 (1.3) | 0 (0-4) | 0 (0-1) | 1.6 (1.5) | 0 (0-4) | 1 (0-3) |  |
| 22. Does not care to upset | 1 (1.3) | 0 (0-4) | 0 (0-2) | 2.4 (1.5) | 4 (0-4) | 2 (1-4) |  |
| 23 Stressed or upset about past | 1.5 (1.4) | 0 (0-4) | 1 (0-2.8) | 1.3 (1.5) | 0 (0-4) | 1 (0-2) |  |
| 24. Refuse to follow rules | 1.2 (1.2) | 0 (0-4) | 1 (0-2) | 1.1 (1.3) | 0 (0-4) | 1 (0-2) |  |
| 25. Senses seem to bother | 2 (1.5) | 0 (0-4) | 2 (0.3-3) | 2.1 (1.6) | 4 (0-4) | 2 (1-4) |  |
| 26. Hit or hurt people | 0.6 (1.1) | 0 (0-4) | 0 (0-1) | 0.2 (0.7) | 0 (0-4) | 0 (0-0) |  |
| 27. Aches, pains and/or lack energy | 1.3 (1.4) | 0 (0-4) | 1 (0-2) | 0.6 (1.3) | 0 (0-4) | 0 (0-0) |  |
| 28. Sexual behaviours bother others | 0.1 (0.5) | 0 (0-4) | 0 (0-0) | 0.3 (0.8) | 0 (0-3) | 0 (0-0) |  |
| 29. Scared of animals or situations | 1.8 (1.6) | 0 (0-4) | 2 (0-3) | 1.4 (1.6) | 0 (0-4) | 1 (0-3) |  |
| 30. Setting fire to things | 0.4 (0.9) | 0 (0-4) | 0 (0-0) | 0.9 (1.3) | 0 (0-4) | 0 (0-2) |  |
| 31. Worry about getting fat | 0.8 (1.3) | 0 (0-4) | 0 (0-1) | 0.6 (1.1) | 0 (0-4) | 0 (0-1) |  |
| 32. Enjoy hurting people or animals | 0.2 (0.5) | 0 (0-3) | 0 (0-0) | 0.4 (0.9) | 0 (0-4) | 0 (0-0) |  |
| 33. Eat too much | 0.7 (1.1) | 0 (0-4) | 0 (0-1) | 0.4 (1.2) | 0 (0-4) | 0 (0-0) |  |
| 34. Ritual that must do to stop feeling upset | 1.4 (1.6) | 0 (0-4) | 1 (0-3) | 1.5 (1.5) | 0 (0-4) | 1 (0-2.5) |  |
| 35. Drugs or alcohol | 0.1 (0.5) | 0 (0-3) | 0 (0-0) | 1.4 (1.5) | 0 (0-4) | 1 (0-3) |  |
| 36. See or hear things that others cannot | 0.6 (1.1) | 0 (0-4) | 0 (0-1) | 0.8 (1.4) | 0 (0-4) | 0 (0-1) |  |
| 37.Think about killing him/herself | 0.5 (0.9) | 0 (0-4) | 0 (0-1) | 0.5 (1) | 0 (0-4) | 0 (0-1) |  |
| 37b.Has tried to kill him/herself | 1 (1.4) | 0 (0-4) | 0 (0-2) | 0.5 (1) | 0 (0-4) | 0 (0-0) |  |
| 38. Extremely happy or excited all the time | 1.2 (1.3) | 0 (0-4) | 1 (0-2) | 1.8 (1.5) | 0 (0-4) | 2 (0-3) |  |
| 39. Hard to wake up sleepy during the day | 1.8 (1.4) | 0 (0-4) | 2 (1-3) | 1.2 (1.4) | 0 (0-4) | 1 (0-2) |  |
| 40. Control people | 0.6 (1) | 0 (0-4) | 0 (0-1) | 2.1 (1.5) | 4 (0-4) | 2 (1-4) |  |
| 41. Look after self, less | 1 (1.3) | 0 (0-4) | 0 (0-2) | 2 (1.4) | 2 (0-4) | 2 (1-3) |  |
| 42. People force to do things that doesn’t want | 0.2 (0.7) | 0 (0-4) | 0 (0-0) | 0.2 (0.7) | 0 (0-4) | 0 (0-0) |  |
| 43. Dislike being separated from certain people | 1.5 (1.5) | 0 (0-4) | 1 (0-3) | 1.5 (1.5) | 0 (0-4) | 1 (0-3) |  |
| 44. Do things that knows shouldn't, to get attention | 0.9 (1.2) | 0 (0-4) | 0 (0-1) | 0.9 (1.2) | 0 (0-4) | 0 (0-1) |  |
| 45. Think and behave in set way | 2.2 (1.5) | 4 (0-4) | 2 (1-4) | 2.2 (1.5) | 4 (0-4) | 2 (1-4) |  |
| 46. Does things without thinking | 1.5 (1.3) | 0 (0-4) | 1 (0-2) | 1.5 (1.3) | 0 (0-4) | 1 (0-2) |  |
| *item not included in child version, m=min, M=Max, Q1=25%, Q3=75% percentiles | | | | | | | |

| Supplementary Table 6: Item level test-retest reliability (stability in time) for the different versions of the ACB scale | | | | | | |
| --- | --- | --- | --- | --- | --- | --- |
| ACB item | Parents  (N=121) | | Young adults and adolescents  (N=39) | | children  (N=22) | |
|  | %  agreement | weighted  Kappa | %  agreement | weighted  Kappa | %  agreement | weighted  Kappa |
| 1. Part of body that hurts or itches | 87.0 | 0.48 | 88.6 | 0.58 | 81.0 | 0.27 |
| 2. Say bad things to/refuse to talk because of look | 94.1 | 0.63 | 96.3 | 0.51 | 97.2 | 0.75 |
| 3. Nightmares | 94.4 | 0.66 | 95.7 | 0.56 | 92.3 | 0.50 |
| 4. Things that likes to repeat | 89.1 | 0.61 | 90.1 | 0.64 | 80.7 | 0.38 |
| 5. Dislike him/herself | 91.9 | 0.66 | 94.6 | 0.63 | 84.7 | 0.17 |
| 6. Movements speeded up or slowed down | 88.2 | 0.37 | 89.9 | 0.41 |  |  |
| 7. Very interested and think about a lot of time | 83.0 | 0.42 | 86.9 | 0.51 | 79.8 | 0.37 |
| 8. Spend a lot of the day worried | 92.7 | 0.72 | 92.5 | 0.74 | 94.3 | 0.68 |
| 9. Do not acceptable things on the internet | 97.0 | 0.49 | 93.7 | 0.43 | 93.2 | -0.05 |
| 10. Hurt or injure | 92.9 | 0.68 | 93.8 | 0.49 | 84.9 | 0.24 |
| 11. Changes in mood that last | 92.5 | 0.60 | 94.9 | 0.66 | 90.9 | 0.37 |
| 12. Mood changes very quickly | 92.5 | 0.74 | 92.8 | 0.73 | 72.2 | 0.09 |
| 13. Damage items | 93.3 | 0.73 | 95.4 | 0.71 | 91.5 | 0.54 |
| 14. Too much energy | 92.7 | 0.77 | 94.9 | 0.82 | 80.7 | 0.40 |
| 15. Scared when people that don’t know | 91.3 | 0.71 | 93.3 | 0.77 | 80.4 | 0.35 |
| 16. Short attention span | 92.0 | 0.68 | 90.1 | 0.62 | 82.7 | 0.45 |
| 17. Trouble with the police | 98.6 | -0.01 | 95.7 | -0.04 |  |  |
| 18. Stopped enjoying things or lost interest | 90.5 | 0.51 | 88.5 | 0.21 | 81.5 | -0.09 |
| 19. Hard to be happy with self or other people | 88.2 | 0.57 | 91.2 | 0.55 |  |  |
| 20. Thoughts and beliefs which are not real | 92.7 | 0.55 | 91.8 | 0.68 | 83.3 | 0.22 |
| 21. Shout at or threaten | 91.1 | 0.69 | 92.0 | 0.44 | 83.8 | 0.45 |
| 22. Does not care to upset | 88.2 | 0.57 | 92.0 | 0.47 | 84.9 | 0.49 |
| 23 Stressed or upset about past | 90.7 | 0.67 | 90.9 | 0.64 | 84.1 | 0.27 |
| 24. Refuse to follow rules | 91.8 | 0.72 | 94.6 | 0.63 | 80.1 | 0.37 |
| 25. Senses seem to bother | 89.9 | 0.62 | 90.7 | 0.72 | 83.5 | 0.48 |
| 26. Hit or hurt people | 92.8 | 0.72 | 92.8 | 0.42 | 88.4 | 0.60 |
| 27. Aches, pains and/or lack energy | 88.1 | 0.53 | 90.4 | 0.58 | 91.5 | 0.68 |
| 28. Sexual behaviors bother others | 96.2 | 0.53 | 94.9 | 0.00 |  |  |
| 29. Scared of animals or situations | 90.5 | 0.69 | 92.6 | 0.76 | 76.7 | 0.23 |
| 30. Setting fire to things | 98.5 | 0.67 | 98.6 | 0.85 | 96.0 | 0.39 |
| 31. Worry about getting fat | 93.2 | 0.51 | 94.4 | 0.70 | 84.7 | 0.45 |
| 32. Enjoy hurting people or animals | 99.2 | 0.84 | 97.4 | 0.00 | 94.9 | 0.45 |
| 33. Eat too much | 94.3 | 0.80 | 95.7 | 0.69 | 78.7 | 0.44 |
| 34. Ritual that must do to stop feeling upset | 89.9 | 0.56 | 87.2 | 0.59 | 88.4 | 0.42 |
| 35. Drugs or alcohol | 99.2 | 0.00 | 100.0 | 1.00 |  |  |
| 36. See or hear things that others cannot | 95.5 | 0.53 | 96.0 | 0.73 | 95.2 | 0.76 |
| 37.Think about killing him/herself | 97.4 | 0.63 | 95.3 | 0.53 | 96.6 | 0.63 |
| 37b.Has tried to kill him/herself | 93.3 | 0.69 | 81.5 | 0.09 | 87.5 | 0.67 |
| 38. Extremely happy or excited all the time | 92.2 | 0.62 | 92.3 | 0.64 | 88.6 | 0.54 |
| 39. Hard to wake up sleepy during the day | 93.0 | 0.69 | 91.0 | 0.67 | 77.0 | 0.13 |
| 40. Control people | 88.6 | 0.58 | 91.7 | 0.52 | 78.7 | 0.18 |
| 41. Look after self, less | 90.9 | 0.51 | 89.3 | 0.48 | 97.4 | 0.80 |
| 42. People force to do things that doesn’t want | 93.9 | 0.36 | 96.6 | 0.60 | 84.1 | 0.14 |
| 43. Dislike being separated from certain people | 92.7 | 0.72 | 89.3 | 0.60 | 80.7 | 0.16 |
| 44. Do things that knows shouldn't, to get attention | 89.7 | 0.57 | 91.7 | 0.25 | 88.4 | 0.41 |
| 45. Think and behave in set way | 89.8 | 0.62 | 84.9 | 0.45 | 84.4 | 0.43 |
| 46. Does things without thinking | 88.8 | 0.64 | 88.9 | 0.63 | 80.1 | 0.33 |

| **Supplementary Table 7: Three factor EFA solution** | | | |
| --- | --- | --- | --- |
| **ITEM26** | **0.85** |  |  |
| **ITEM24** | **0.80** |  |  |
| **ITEM13** | **0.80** |  |  |
| **ITEM46** | **0.74** |  | -0.29 |
| **ITEM32** | **0.71** |  |  |
| **ITEM21** | **0.68** |  |  |
| **ITEM14** | **0.65** |  | -0.31 |
| **ITEM12** | **0.62** |  |  |
| **ITEM40** | **0.60** |  |  |
| **ITEM22** | **0.55** |  |  |
| **ITEM16** | **0.51** |  |  |
| **ITEM10** | **0.47** | 0.31 |  |
| **ITEM33** | **0.45** |  |  |
| **ITEM34** |  | **0.71** |  |
| **ITEM15** |  | **0.70** |  |
| **ITEM45** |  | **0.63** |  |
| **ITEM43** |  | **0.62** |  |
| **ITEM8** |  | **0.61** | **0.37** |
| **ITEM29** |  | **0.59** |  |
| **ITEM25** |  | **0.52** |  |
| **ITEM27** |  | **0.48** | **0.38** |
| **ITEM1** |  | **0.45** |  |
| ITEM5 |  | 0.33 | **0.61** |
| ITEM9 | 0.37 |  | **0.57** |
| ITEM31 |  | **0.31** | **0.53** |
| ITEM39 |  |  | **0.52** |
| ITEM42 |  |  | **0.47** |
| ITEM20 |  | **0.42** | **0.45** |
| ITEM18 |  |  | **0.43** |
| ITEM19 |  | **0.43** | 0.40 |
| ITEM36 |  | **0.43** |  |
| ITEM3 |  | **0.42** |  |
| ITEM28 | 0.34 |  |  |
| ITEM6 | **0.36** | **0.27** |  |
| ITEM7 |  | **0.41** |  |
| primary loadings are marked with bold, cross loading below 0.3 are not presented | | | |

| **Supplementary Table 8: Descriptive indices per participant sex and in the total sample** | | | | |
| --- | --- | --- | --- | --- |
|  | Females  Mean (SD) | Males  Mean (SD) | Independent samples  t-test | Total sample  Mean (SD) |
| Internalizing | 32.3 (14.6) | 25 (13.7) | *t*=3.487, *df*=249, *p*=0.001 | 26.7 (14.2) |
| Externalizing | 22.4 (14.7) | 22.3 (12.4) | *t*=0.010, *df*=249, *p*=0.992 | 22.4 (13.0) |
| Total ACB | 54.6 (25.8) | 47.4 (22.6) | *t*=2.077, *df*=249, *p*=0.039 | 49.1 (23.6) |

| **Supplementary Table 9: Descriptive indices and alpha values per DQ group** | | | | |
| --- | --- | --- | --- | --- |
|  | | **No DQ**  **(N=52)** | **DQ<70**  **(N=105)** | **DQ≥70**  **(N=98)** |
| **Internalizing** | alpha | 0.88 | 0.84 | 0.87 |
|  | Mean (SD) | **^+^**31.1 (15.4) | **^+^**25.55 (13.3) | **^+^**25.54 (14.1) |
|  | Median (min-max) | 31 (4-67) | 25 (4-63) | 25 (1-63) |
| **Externalizing** | alpha | 0.90 | 0.85 | 0.87 |
|  | Mean (SD) | **^+,‡^**21.96 (13.9) | **^+^**25.49 (12.6) | **^‡^**19.27 (12.3) |
|  | Median (min-max) | 21 (0-56) | 26 (2-52) | 19 (0-50) |
| **Total ACB** | alpha | 0.93 | 0.87 | 0.92 |
|  | Mean (SD) | **^+^**53.06 (27.1) | **^+^**51.04 (20.9) | **^+^**44.81 (23.9) |
|  | Median (min-max) | 54.5 (6-121) | 50 (9-100) | 43 (2-95) |
| Means with the same superscript within row do not differ significantly (Bonferroni adjusted p>0.05) | | | | |

|  | | **Supplementary Table 10: Pearson Correlation coefficients between the ACB- parent scores and other measures per DQ group** | | | | | | | | |
| --- | --- | --- | --- | --- | --- | --- | --- | --- | --- | --- |
|  | | **No DQ estimate** | | | **DQ<70** | | | **DQ>70** | | |
|  |  | **ACB Internalizing** | **ACB Externalizing** | **Total ACB** | **ACB Internalizing** | **ACB Externalizing** | **Total ACB** | **ACB Internalizing** | **ACB Externalizing** | **Total ACB** |
|  | | *n=52* | | | *n=105* | | | *n=98* | | |
| **ACB** | **Externalizing** | **^+^**0.71** |  | **^+^**0.92* | ***^‡^0.30***** |  | ***^‡^0.80***** | **^+^**0.63** |  | **^+^**0.89** |
|  | **Total** | **^+^**0.93** | **^+^**0.92** |  | ***^‡^0.82***** | ***^‡^0.80***** |  | **^+^**0.92** | **^+^**0.89** |  |
|  | | *n=37* | | | *n=81* | | | *n=75* | | |
| **ABC** | **Irritability** | **^+^**0.62** | **^+, ‡^** 0.79** | **^+^**0.76** | ^+^0.44** | **^‡^*0.74***** | ^+^0.73** | **^+^**0.61** | **^+^**0.84** | **^+^**0.78** |
|  | **Lethargy** | **^+^**0.63** | **^+^**0.53** | **^+^**0.64** | ^+^0.60** | ^+^0.30** | ^+^0.56** | **^+^**0.57** | **^+^**0.44** | **^+^**0.56** |
|  | **Stereotypy** | **^+^**0.40* | **^+^**0.24 | **^+^**0.36* | ***^‡^0.12*** | ^+^0.37** | ^+^0.31** | **^+^**0.48** | **^+^**0.38** | **^+^**0.47** |
|  | **Hyperactivity** | **^+^**0.49** | **^+^**0.67** | **^+^**0.62** | ***^‡^0.12*** | ^+^0.80** | ^+^0.55* | **^+^**0.49** | **^+^**0.74** | **^+^**0.66** |
|  | **Speech** | **^+^**0.33* | **^+^**0.42* | **^+^**0.40* | ^+^0.12 | ^+^0.19 | ^+^0.19 | **^+^**0.34** | **^+^**0.29* | **^+^**0.35** |
|  | **Total ABC** | **^+^**0.67** | **^+^**0.76** | **^+^**0.77* | ***^‡^0.41***** | 0.74** | 0.71** | **^+^**0.66** | **^+^**0.76** | **^+^**0.77** |
|  | | *n=41* | | | *n=91* | | | *n=85* | | |
| **MOAS** | **Verbal aggression** | **^+^**0.31 | **^+^**0.53** | **^+^**0.45** | **^+^**0.26* | **^+^**0.50** | **^+^**0.44** | +0.24* | +0.56** | +0.42** |
|  | **Aggression against property** | **^+^**0.35* | **^+^**0.59** | **^+^**0.51** | **^+^**0.24* | **^+^**0.61** | **^+^**0.53** | +0.26* | +0.58** | +0.45** |
|  | **Auto aggression** | **^+^**0.30 | **^+^**0.57** | **^+^**0.46** | **^+^**0.28** | **^+^**0.53** | **^+^**0.49** | +0.28* | ***^‡^0.29***** | +0.31** |
|  | **Physical aggression** | **^+^**0.21 | **^+^**0.59** | **^+^**0.42** | **^+^**0.06 | **^+^**0.56** | **^+^**0.38** | +0.23* | +0.52** | +0.38** |
|  | **Total MOAS** | **^+^**0.35* | **^+^**0.68** | **^+^**0.54** | **^+^**0.25* | **^+^**0.68** | **^+^**0.57** | +0.30** | +0.59** | +0.48** |
|  | | *n=40* | | | *n=86* | | | *n=85* | | |
| **ASEBA** | **Total ASEBA** | **^+,^*^‡^***0.79** | **^+^**0.74** | **^+^**0.84** | ***^‡^0.67***** | **^+^**0.62** | **^+^**0.80** | **^+^**0.79** | **^+^**0.83** | ***^‡^0.88***** |
|  | **ASEBA Internalizing** | **^+^**0.77* | **^+^**0.53** | **^+^**0.73** | **^+^**0.78** | ***^‡^0.18*** | **^+^**0.61** | **^+^**0.80** | **^+^**0.59** | ***^‡^0.76***** |
|  | **ASEBA Externalizing** | **^+^**0.58** | **^+^**0.83** | **^+^**0.75** | ***^‡^0.30***** | **^+^**0.79** | **^+^**0.66** | **^+^**0.58** | **^+^**0.87** | **^+^**0.78** |
|  | | *n=46* | | | *n=98* | | | *n=94* | | |
| **SCQ** | **Total SCQ** | **^+^**0.24 | **^+^**0.35* | **^+^**0.32* | **^+^**0.23* | **^+^**0.26* | **^+^**0.30* | **^+^**0.35** | **^+^**0.24* | **^+^**0.33** |
|  | | *n=39* | | | *n=71* | | | *n=77* | | |
| **HSQ** | **Social Flexibility** | **^+,^*^‡^***0.56** | **^+^**0.72** | **^+^**0.71** | **^+^**0.29* | **^+^**0.65** | **^+^**0.59** | ***^‡^0.53***** | **^+^**0.58** | **^+^**0.61** |
|  | **Demand Specific** | **^+^**0.57** | **^+^**0.72** | **^+^**0.71** | **^+^**0.33** | **^+^**0.71** | **^+^**0.65** | **^+^**0.50** | **^+^**0.66** | **^+^**0.63** |
|  | **Total HSQ** | **^+^**0.58** | **^+^**0.74** | **^+^**0.73** | **^+^**0.33** | **^+^**0.72** | **^+^**0.65** | **^+^**0.53** | **^+^**0.65** | **^+^**0.64** |
| Correlations coefficients which represent the same relationship across groups, which share the same **^+ ‡^**superscript did not differ significantly (p>0.05). Significantly different coefficients denoted with bold italics. | | | | | | | | | | |
